# Comparative effectiveness and outcomes of physiology- and imaging-guided PCI: an evidence synthesis and network meta-analysis of FFR, iFR, OCT, and IVUS

**DOI:** 10.3389/fcvm.2026.1762634

**Published:** 2026-03-20

**Authors:** Yuancheng Jin, Heyong Liu, Heng Gan, Bin He, Wei Wang, Xin Gan, Lin Hong, Harshawardhan Dhanraj Ramteke, LiPing Luo, Zhicheng Huang

**Affiliations:** 1 Department of Pharmacy, the Fourth Affiliated Hospital of School of Medicine, and International School of Medicine, International Institutes of Medicine, Zhejiang University, Yiwu, Zhejiang, China; 2 Department of Pharmacy, Ezhou Central Hospital, Ezhou, Hubei, China; 3 Department of Pharmacy, Luzhou Hospital of Traditional Chinese Medicine, Luzhou, Sichuan, China; 4 Department of Pharmacy, Chongren County Hospital of Traditional Chinese Medicine, Fuzhou, Jiangxi, China; 5 Department of Pharmacy, Luxian County Second People’s Hospital, Luzhou, Sichuan, China; 6 Department of Cardiology, Anhui Medical University, Hefei, Anhui, China

**Keywords:** instantaneous wave free ratio (iFR), fractional flow reserve (FFR), intravascular ultrasound (IVUS), optical coherence tomography (OCT), percutaneous coronary intervention (PCI)

## Abstract

**Background:**

Multiple coronary guidance strategies including angiography, physiology-based assessment, and intracoronary imaging are used to optimize percutaneous coronary intervention, yet their comparative effectiveness across clinical outcomes remains uncertain.

**Methods:**

A comprehensive network meta-analysis incorporated fifty randomized studies evaluating angiography, FFR, iFR, IVUS, and OCT. The primary outcome was major adverse cardiovascular events (MACE). Secondary outcomes included all-cause mortality, cardiac death, myocardial infarction, stent thrombosis, target lesion revascularization, and target vessel revascularization. Random effects models were applied and interventions were ranked using SUCRA.

**Results:**

A total of 50 studies involving 39,863 patients were included, of whom 29,571 were male and 10,031 were female. Across guidance modalities, 15,463 patients underwent angiography-guided PCI, 10,728 IVUS-guided, 6,001 FFR-guided, 3,512 iFR-guided, and 3,849 OCT-guided PCI. In the network meta-analysis, intravascular imaging strategies demonstrated favorable outcomes across evaluated endpoints. Compared with IVUS, angiography-guided PCI was associated with higher rates of major adverse cardiovascular events (RR 1.28, 95% CI 1.13–1.46), all-cause mortality (RR 1.30, 95% CI 0.98–1.63), myocardial infarction (RR 1.73, 95% CI 1.28–2.40), target lesion failure (RR 1.50, 95% CI 1.19–1.93), and stent thrombosis (RR 1.80, 95% CI 1.25–2.70). Physiology-guided PCI using iFR was associated with higher risk estimates for all-cause mortality (RR 1.72, 95% CI 1.06–2.79) and cardiac death (RR 2.21, 95% CI 1.24–4.24) compared with IVUS. OCT demonstrated outcomes comparable to IVUS, with no statistically significant differences in major adverse cardiovascular events (RR 1.00, 95% CI 0.80–1.28) or cardiac death (RR 0.86, 95% CI 0.47–1.59). Sensitivity analyses yielded similar estimates. Overall, probabilistic ranking analyses favored intravascular imaging strategies, although effect estimates among non-angiographic modalities overlapped.

**Conclusions:**

Advanced PCI guidance strategies using intravascular imaging or invasive physiological assessment are associated with improved clinical outcomes compared with angiography alone. However, no single non-angiographic modality demonstrates definitive superiority, supporting individualized selection of guidance strategies based on clinical and procedural context.

**Systematic Review Registration:**

https://www.crd.york.ac.uk/PROSPERO/view/CRD420251238909, identifier CRD420251238909.

## 1 Introduction

Percutaneous coronary intervention (PCI) remains a cornerstone of contemporary management for coronary artery disease, yet the optimal strategy to guide lesion assessment, stent implantation, and post-procedural optimization continues to evolve (1). Traditional angiography provides a two-dimensional luminogram that often inadequately reflects the physiological significance of coronary stenoses or the structural integrity of stent deployment (2). Recent trials in chronic coronary syndromes have underscored the limitations of angiography alone, demonstrating that more nuanced, patient-specific approaches are required to achieve meaningful clinical benefit (3, 4).

Two complementary paradigms have emerged to refine PCI: **coronary physiology** and **intravascular imaging**. Physiology-based techniques such as fractional flow reserve (FFR) and instantaneous wave-free ratio (iFR) allow objective assessment of ischemia and inform treatment vs. deferral of intermediate coronary lesions (5). These approaches reduce unnecessary stenting, lower procedural risk, and are endorsed by current guidelines as evidence-based determinants of revascularization appropriateness (6). Additionally, post-PCI physiologic indices have been consistently associated with long-term outcomes, with suboptimal values predicting increased risk of myocardial infarction and target vessel failure (7).

Intravascular imaging modalities, including intravascular ultrasound (IVUS) and optical coherence tomography (OCT), provide unprecedented visualization of plaque morphology, vessel size, stent expansion, and healing (8). Imaging-guided PCI has been shown to identify mechanical complications—such as stent underexpansion, malapposition, and edge dissection, that are not apparent on angiography but strongly linked to restenosis and stent thrombosis (9). These modalities enhance procedural precision and have been associated with improved device-oriented and patient-oriented outcomes across diverse lesion and patient subsets (10).

Despite robust evidence supporting both physiology-guided and imaging-guided strategies, comparative data across all modalities remain limited (11). Most randomized trials have relied on pairwise comparisons, and no single study has simultaneously evaluated all guidance methods. As a result, the relative benefits of FFR, iFR, IVUS, and OCT remain unclear, and clinicians lack a unified evidence base to inform optimal guidance selection in daily practice (12). A comprehensive synthesis that integrates direct and indirect evidence across available randomized trials is therefore essential.

In this context, we performed a systematic review and network meta-analysis to compare the clinical effectiveness of physiology-guided and intravascular imaging-guided PCI strategies. By evaluating outcomes across the full spectrum of contemporary guidance modalities, this study aims to establish a comparative effectiveness hierarchy and provide clarity on the optimal strategy for improving clinical outcomes in patients undergoing PCI.

## 2 Methods

### 2.1 Literature search

A comprehensive literature search was performed across major electronic databases, including PubMed, Embase, Scopus, Web of Science, and the Cochrane Library. Search terms incorporated combinations of keywords related to PCI guidance strategies, such as “percutaneous coronary intervention,” “fractional flow reserve,” “instantaneous wave-free ratio,” “intravascular ultrasound,” “optical coherence tomography,” and “angiography-guided PCI.” The search included randomized controlled trials (RCTs) published in English up to December 2025, ensuring the capture of all contemporary evidence reflecting modern physiological and intravascular imaging practices. Study identification, screening, and data extraction followed the PRISMA (Preferred Reporting Items for Systematic Reviews and Meta-Analyses) guidelines to ensure transparency, methodological rigor, and reproducibility (13). The protocol for this systematic review and network meta-analysis was prospectively registered with PROSPERO (Registration ID: CRD420251238909) (14).

### 2.2 Study selection and data extraction

The study selection followed a predefined protocol with explicit eligibility criteria. All records identified through database searches were imported into EndNote for de-duplication. Titles and abstracts were screened to exclude studies not evaluating percutaneous coronary intervention (PCI) guidance strategies, and full-text articles of potentially eligible studies were independently reviewed by two investigators. Studies were eligible if they were randomized controlled trials directly comparing at least two PCI guidance strategies—angiography, fractional flow reserve (FFR), instantaneous wave-free ratio (iFR), intravascular ultrasound (IVUS), or optical coherence tomography (OCT); used the assigned modality as the randomized strategy for PCI decision-making and/or procedural optimization; and reported clinically relevant outcomes (e.g., major adverse cardiovascular events, mortality, myocardial infarction, stent thrombosis, or target lesion/vessel revascularization) with sufficient data for extraction. Studies were excluded if they were non-randomized, observational, diagnostic, or *post-hoc* analyses; did not involve PCI or evaluated non-coronary interventions; used imaging or physiological assessments only adjunctively or retrospectively rather than as the randomized guidance strategy; evaluated angiography-derived computational physiological indices; or lacked extractable outcome data. Data were independently extracted by two reviewers using a standardized, pilot-tested form. Extracted information included study characteristics, patient and lesion features, details of the PCI guidance strategy, and all prespecified clinical outcomes. Disagreements were resolved by consensus or adjudication by a third reviewer.

### 2.2 Risk of bias assessment

The risk of bias for all included randomized controlled trials was evaluated using the **Cochrane Risk of Bias 2 (RoB 2)** tool (15). Domains assessed included randomization process, deviations from intended interventions, missing outcome data, outcome measurement, and selective reporting. Each domain and the overall study were categorized as **low risk**, **some concerns**, or **high risk**. The risk-of-bias assessment informed the certainty of evidence and sensitivity analyses.

### 2.3 Statistical analysis

A random-effects model was applied for both pairwise and network meta-analyses to account for anticipated clinical and methodological heterogeneity across trials. Dichotomous outcomes were synthesized using risk ratios (RRs) with corresponding 95% confidence intervals, while continuous outcomes were summarized using mean differences. A Bayesian network meta-analysis was performed to integrate direct and indirect evidence across all PCI guidance strategies (angiography, fractional flow reserve [FFR], instantaneous wave-free ratio [iFR], intravascular ultrasound [IVUS], and optical coherence tomography [OCT]), with model convergence assessed using standard diagnostics. IVUS was selected as the reference comparator in the network meta-analysis because it represents the most extensively studied intravascular imaging modality in randomized PCI trials and provides the greatest network connectivity across direct and indirect comparisons. This approach improves model stability and interpretability of relative treatment effects without implying clinical superiority. Between-study heterogeneity was quantified using the posterior estimate of the between-study variance (*τ*²) within the random-effects framework. Posterior treatment rankings were derived using surface under the cumulative ranking curve (SUCRA) probabilities and Bayesian rankograms. Sensitivity analyses were conducted by excluding studies at high risk of bias. Predefined subgroup network meta-analyses were not performed due to limited data within individual network nodes and the risk of network fragmentation and ecological bias. Publication bias was assessed through visual inspection of funnel plots and, where applicable, statistical tests for small-study effects. All analyses were conducted using R software, employing the *gemtc*, *rjags*, and related Bayesian network meta-analysis packages.

### 2.4 Outcomes and definitions

Major adverse cardiovascular events (MACE) were prespecified as the primary outcome. Secondary outcomes included all-cause mortality, cardiac death, myocardial infarction, stent thrombosis, and target lesion or target vessel revascularization. MACE was defined according to the definitions used in the individual included trials. As the components of MACE varied across studies (e.g., death, myocardial infarction, repeat revascularization, and/or stroke), trial-specific definitions were extracted and analyzed as reported, without redefinition or harmonization. Definitions of MACE, modality used for decision making and PCI optimization, used in each study are summarized in Supplementary Table S1.

## 3 Results

### 3.1 Demographics

**Figure 1:**
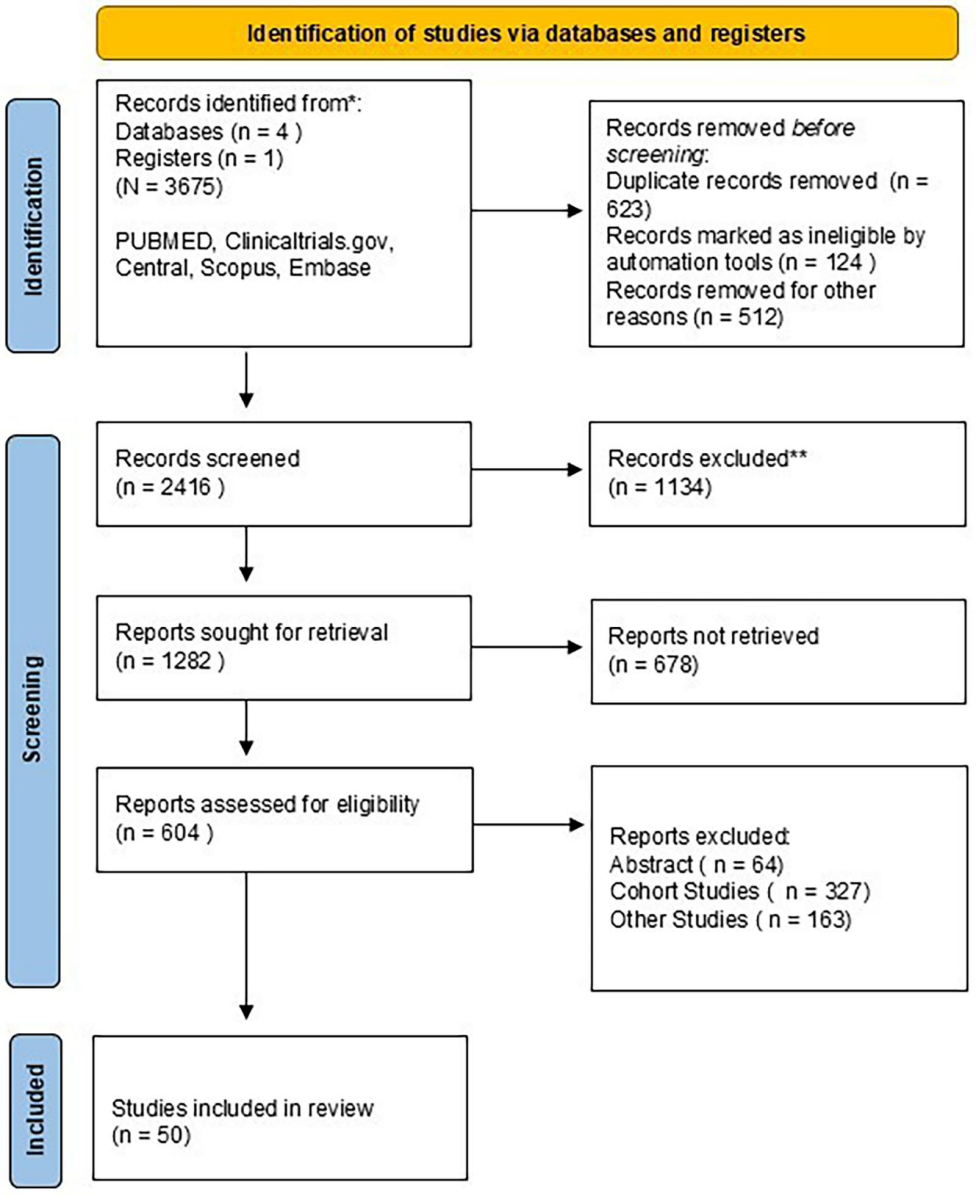
PRISMA flow diagram.

A total of 3,765 studies were identified through the initial search, of which 50 trials (16–64) fulfilled the eligibility criteria and were included in the final analysis (Figure 1). Across these studies, 39,863 participants were enrolled, comprising 29,571 men and 10,031 women, with a mean age of 62.35 ± 10.87 years and a mean follow-up duration of 18.56 ± 14.45 months. In total, 19,860 patients were assigned to treatment groups and 19,693 to control groups, contributing 79,726 pooled observations for baseline characterization. The network encompassed 15,463 angiography-guided, 10,728 IVUS-guided, 6,001 FFR-guided, 3,512 iFR-guided, and 3,849 OCT-guided PCI procedures, forming a robust, well-connected evidence structure suitable for Bayesian network meta-analysis. Baseline clinical characteristics were broadly comparable across treatment and control groups (Supplementary Tables S2, S3). Diabetes was present in 8,534 vs. 8,378, hypertension in 13,656 vs. 13,516, hyperlipidemia in 14,045 vs. 14,927, prior myocardial infarction in 3,826 vs. 3,812, prior PCI in 4,442 vs. 4,290, and chronic kidney disease in 2,126 vs. 2,172 patients, respectively. Presenting syndromes were similarly distributed, including unstable angina (2,730 vs. 2,737), stable angina (1,752 vs. 1,775), STEMI (863 vs. 883), and NSTEMI (1,534 vs. 1,540). Coronary anatomical complexity was likewise well balanced, with single-vessel (5,990 vs. 6,039), double-vessel (4,871 vs. 4,900), triple-vessel (3,286 vs. 3,322), and chronic total occlusion (383 vs. 387) lesions represented in similar proportions. A total of 9,882 vs. 9,857 lesions were treated across groups, including bifurcation (321 vs. 338), left main (272 vs. 281), and small-vessel (623 vs. 651) lesions. Safety outcomes were infrequent across all trials. Heart failure hospitalization occurred in 117 vs. 125 patients, major bleeding in 110 vs. 117, and acute kidney injury in 19 vs. 24, highlighting the overall low procedural risk profile across guidance strategies.

### 3.2 Major adverse cardiovascular events (MACE)

**Figure 2:**
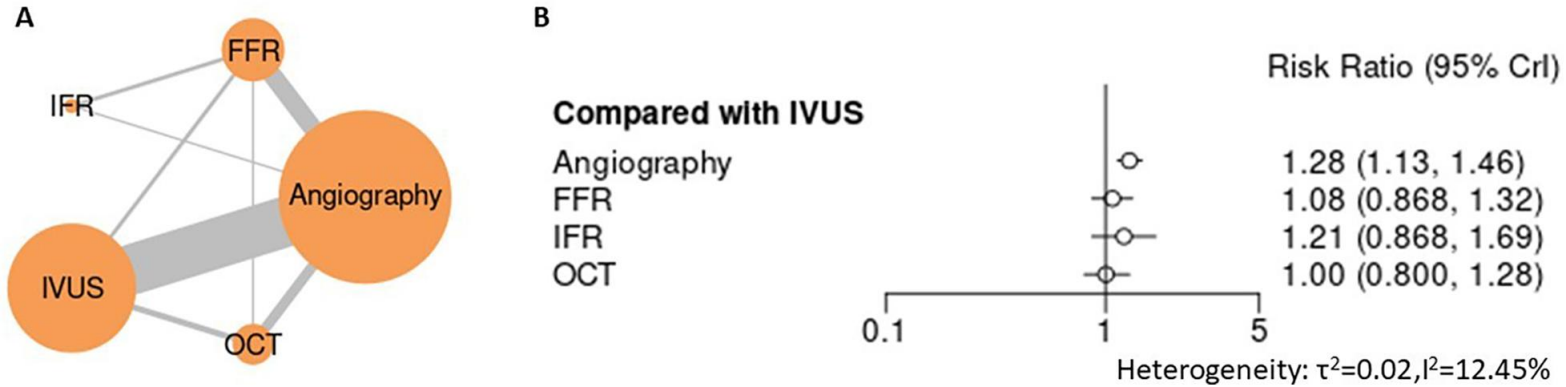
Network meta-analysis of major adverse cardiovascular events (MACE). **(A)** Network plot showing direct and indirect comparisons among PCI guidance strategies. **(B)** Forest plot of pooled relative effect estimates (risk ratios with 95% credible intervals).

Across the network of five PCI guidance strategies, advanced guidance approaches were associated with lower rates of major adverse cardiovascular events compared with angiography-guided PCI. In the primary network meta-analysis (Figure 2, Supplementary Figure S1), angiography-guided PCI was associated with a significantly higher risk of MACE compared with intravascular ultrasound (IVUS) (RR 1.28, 95% CI 1.13–1.46). In contrast, no statistically significant differences in MACE were observed between IVUS and other non-angiographic strategies, including optical coherence tomography (OCT) (RR 1.00, 95% CI 0.80–1.28), fractional flow reserve (FFR) (RR 1.08, 95% CI 0.87–1.32), or instantaneous wave-free ratio (iFR) (RR 1.21, 95% CI 0.87–1.69), with overlapping confidence intervals across comparisons. Between-study heterogeneity was low (*τ*² = 0.02), indicating good network consistency. Sensitivity analyses excluding studies at higher risk of bias yielded directionally consistent findings, with angiography remaining associated with higher MACE risk relative to IVUS (RR 1.32, 95% CI 1.15–1.52) and no material changes in comparisons among non-angiographic strategies (Supplementary Figure S2). Overall, these results demonstrate a clear benefit of advanced PCI guidance over angiography alone, without evidence of definitive superiority among non-angiographic modalities.

### 3.3 Mortality outcomes

**Figure 3:**
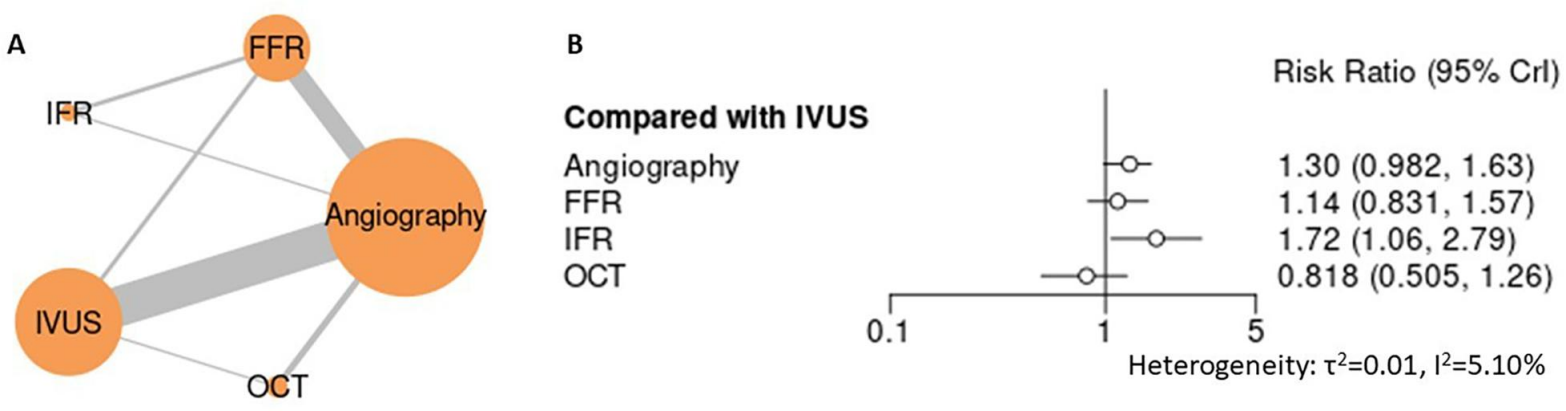
Network meta-analysis of all-cause mortality. **(A)** Network plot depicting direct and indirect comparisons among included interventions for all- cause mortality. **(B)** Forest plot presenting pooled relative effect estimates (risk ratios with 95% CIs) for all pairwise comparisons within the network.

**Figure 4:**
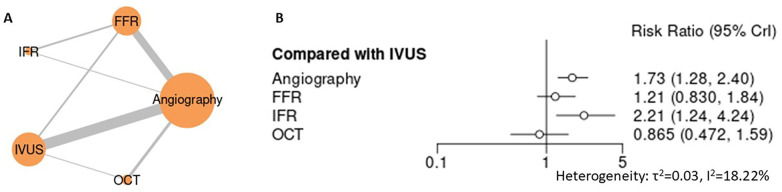
Network meta-analysis of death caused by myocardial infarction. **(A)** Network plot depicting direct and indirect comparisons among included interventions for death caused by myocardial infarction. **(B)** Forest plot presenting pooled relative effect estimates (risk ratios with 95% CIs) for all pairwise comparisons within the network.

Across mortality endpoints, angiography-guided PCI was associated with less favorable outcomes compared with advanced guidance strategies. In the primary analysis of all-cause mortality (Figure 3, Supplementary Figure S3 for league table and SUCRA), angiography demonstrated a numerically higher risk compared with IVUS (RR 1.30, 95% CI 0.98–1.63), while FFR showed a similar nonsignificant trend (RR 1.14, 95% CI 0.83–1.57). iFR-guided PCI was associated with a significantly higher estimate of all-cause mortality relative to IVUS (RR 1.72, 95% CI 1.06–2.79), whereas OCT demonstrated a numerically lower risk (RR 0.82, 95% CI 0.50–1.26), though without statistical significance. For mortality due to myocardial infarction (Figure 4), angiography-guided PCI was associated with a higher risk compared with IVUS (RR 1.73, 95% CI 1.28–2.40), as was iFR-guided PCI (RR 2.21, 95% CI 1.24–4.24). No statistically significant differences were observed between IVUS and either FFR (RR 1.21, 95% CI 0.83–1.84) or OCT (RR 0.87, 95% CI 0.47–1.59). Across mortality analyses, heterogeneity was low to moderate, and sensitivity analyses yielded directionally consistent findings with stable treatment hierarchies (Supplementary Figures S3–S6). Collectively, these results support the superiority of advanced guidance strategies over angiography alone for mortality outcomes, without establishing a single optimal non-angiographic modality.

### 3.4 Device- and lesion-oriented outcomes

**Figure 5:**
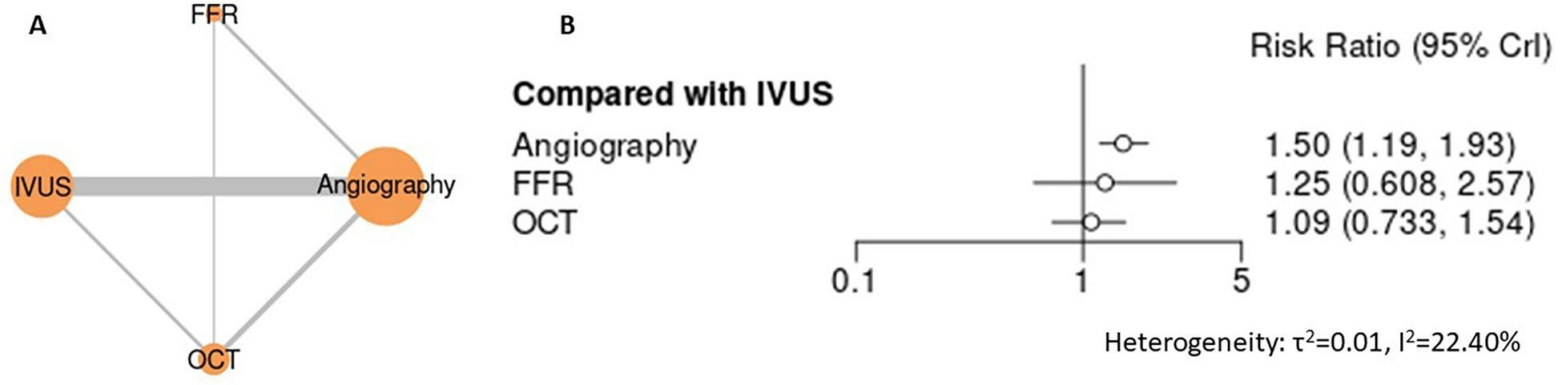
Network meta-analysis of target lesion failure. **(A)** Network plot depicting direct and indirect comparisons among included interventions for target lesion failure. **(B)** Forest plot presenting pooled relative effect estimates (risk ratios with 95% CIs) for all pairwise comparisons within the network.

**Figure 6:**
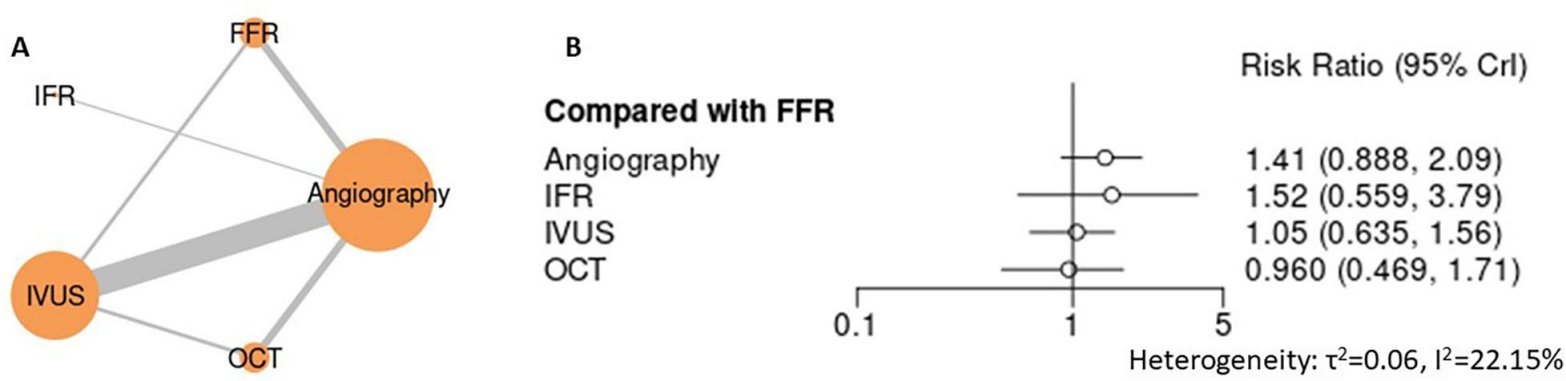
Network meta-analysis of target lesion revascularization (TLR). **(A)** Network plot depicting direct and indirect comparisons among included interventions for target lesion revascularization. **(B)** Forest plot presenting pooled relative effect estimates (risk ratios with 95% CIs) for all pairwise comparisons within the network.

**Figure 7:**
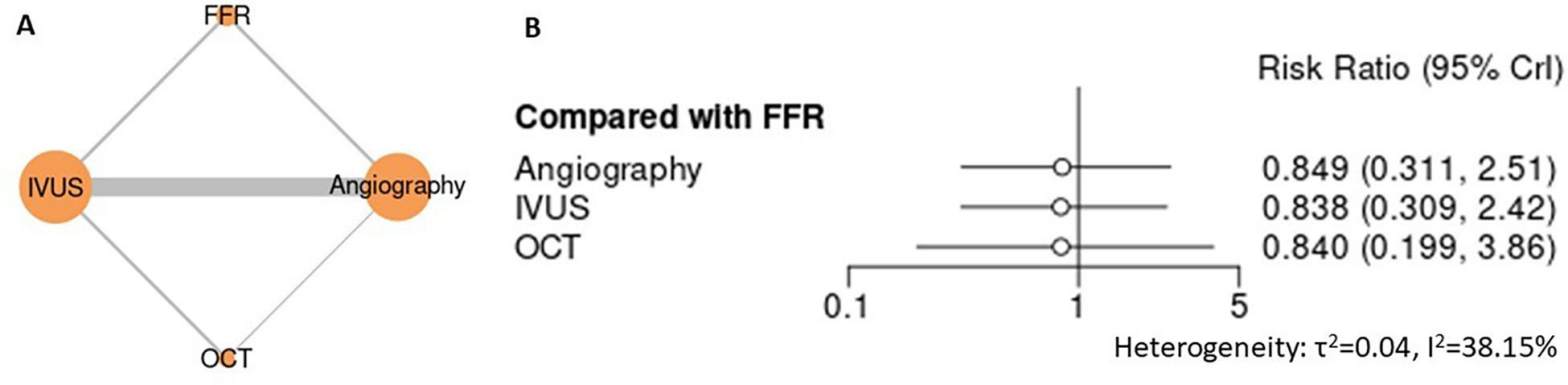
Network meta-analysis of target vessel revascularization (TVR). **(A)** Network plot depicting direct and indirect comparisons among included interventions for target vessel revascularization. **(B)** Forest plot presenting pooled relative effect estimates (risk ratios with 95% CIs) for all pairwise comparisons within the network.

**Figure 8:**
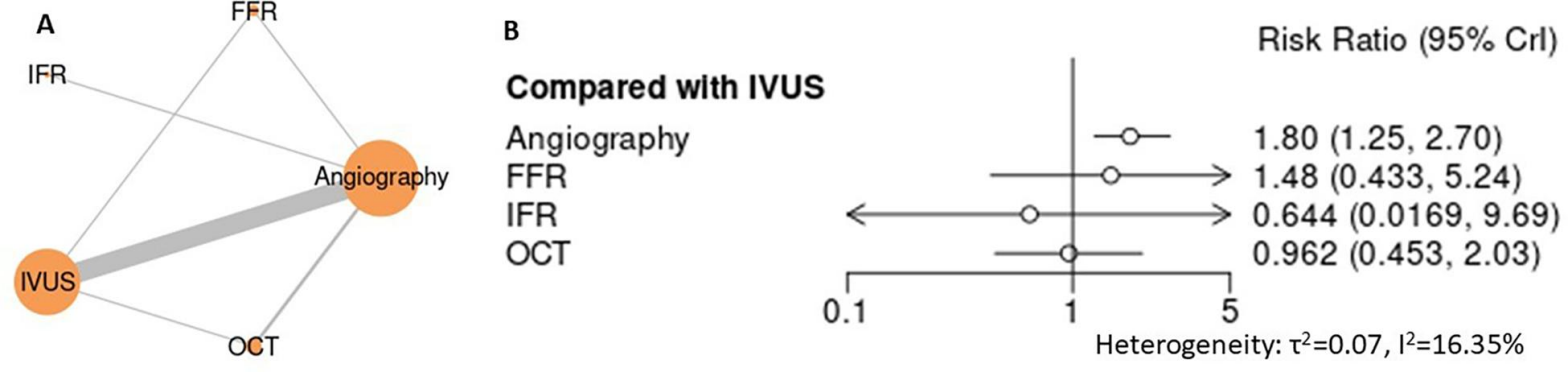
Network meta-analysis of stent thrombosis. **(A)** Network plot depicting direct and indirect comparisons among included interventions for target vessel revascularization. **(B)** Forest plot presenting pooled relative effect estimates (risk ratios with 95% CIs) for all pairwise comparisons within the network.

For lesion- and device-related outcomes, including target lesion failure (TLF), target lesion revascularization (TLR), target vessel revascularization (TVR), and stent thrombosis, intravascular imaging–guided PCI demonstrated consistently favorable profiles compared with angiography-guided PCI (Figures 5–8). In the analysis of target lesion failure (Figure 5, Supplementary Figure S7), angiography-guided PCI was associated with a significantly higher risk compared with IVUS (RR 1.50, 95% CI 1.19–1.93), whereas no statistically significant differences were observed between IVUS and FFR (RR 0.80, 95% CI 0.39–1.64) or OCT (RR 1.09, 95% CI 0.73–1.54). For target lesion revascularization (Figure 6, Supplementary Figure S9), IVUS-guided PCI was associated with a lower risk compared with angiography (RR 0.74, 95% CI 0.57–0.95), while comparisons among IVUS, OCT, and FFR did not demonstrate statistically significant differences. No guidance strategy demonstrated a statistically significant reduction in target vessel revascularization compared with FFR (Figure 7, Supplementary Figure S11), with all confidence intervals crossing unity. In the stent thrombosis analysis (Figure 8, Supplementary Figure S13), angiography-guided PCI was associated with a significantly higher risk compared with IVUS (RR 1.80, 95% CI 1.25–2.70), whereas FFR, iFR, and OCT demonstrated comparable risks relative to IVUS. Across device-related endpoints, heterogeneity was generally low to moderate, and sensitivity analyses confirmed the stability of effect estimates and comparative patterns (Supplementary Figures S8, S10, S12, S14).

### 3.5 Bleeding events

**Figure 9:**
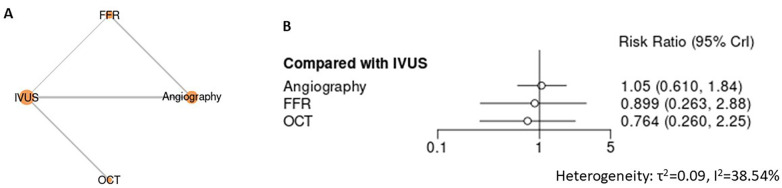
Network meta-analysis of bleeding events. **(A)** Network plot depicting direct and indirect comparisons among included interventions for bleeding events. **(B)** Forest plot presenting pooled relative effect estimates (risk ratios with 95% CIs) for all pairwise comparisons within the network.

No statistically significant differences in bleeding events were observed among angiography-, physiology-, or imaging-guided PCI strategies (Figure 9). Using IVUS as the reference, angiography (RR 1.05, 95% CI 0.61–1.84), FFR (RR 0.90, 95% CI 0.26–2.88), and OCT (RR 0.76, 95% CI 0.26–2.25) demonstrated overlapping estimates, indicating comparable bleeding risk across modalities. Studies were less to perform the sensitivity analysis.

### 3.6 Sensitivity and consistency analyses

Sensitivity analyses excluding studies at higher risk of bias or contributing to heterogeneity yielded directionally consistent results across all primary and secondary outcomes. Relative treatment effects and comparative patterns were preserved, and no violations of transitivity or major network inconsistency were identified. These findings support the robustness and internal validity of the primary network meta-analysis.

### 3.7 Risk of bias

The risk-of-bias evaluation revealed that the methodological quality of the included trials was generally robust. Most studies demonstrated low risk across key domains, including randomization, completeness of outcome data, outcome measurement, and selective reporting, reflecting adherence to rigorous trial conduct standards. A notable exception was the domain assessing deviations from intended interventions, where a proportion of studies exhibited high risk, suggesting potential inconsistencies in protocol adherence or treatment implementation. Nevertheless, the overall risk-of-bias profile remained predominantly low, indicating that the synthesized evidence is derived from studies with acceptable internal validity and minimal methodological concerns.

## 4 Discussion

In this comprehensive network meta-analysis comparing angiographic, physiological, and intravascular imaging guidance strategies for percutaneous coronary intervention, several important observations emerge that refine current understanding of optimal approaches to improve cardiovascular outcomes. Across multiple clinically relevant endpoints including major adverse cardiovascular events, all cause and cardiac mortality, myocardial infarction related outcomes, target lesion failure, target vessel revascularization, stent thrombosis, and bleeding, advanced PCI guidance strategies consistently demonstrated superior outcomes compared with angiography alone. These findings reinforce the limitations of angiographic assessment as a standalone guidance modality and support the routine use of adjunctive physiological or imaging-based strategies in contemporary PCI practice.

The most robust and consistent finding of this analysis is the clear inferiority of angiography guided PCI. Angiography ranked lowest across nearly all ischemic and mortality related outcomes, underscoring its inability to adequately assess functional lesion severity, plaque morphology, stent expansion, and edge complications. This observation is consistent with prior randomized evidence and meta-analytic data and aligns with current guideline recommendations favoring advanced guidance techniques over angiography alone.

Intravascular imaging guided PCI using intravascular ultrasound and optical coherence tomography demonstrated favorable rankings across several outcomes when compared with angiography. These results are concordant with previous pairwise and network meta-analyses. A systematic review of thirteen randomized controlled trials demonstrated that intravascular ultrasound guided PCI significantly reduced major adverse cardiovascular events with a risk ratio of 0.63 and reduced stent thrombosis with a risk ratio of 0.52 compared with angiography guidance (65). That analysis also identified reductions in cardiac mortality, myocardial infarction, target lesion revascularization, and target vessel revascularization, although all-cause mortality did not reach statistical significance. Our findings validate and extend these observations by incorporating additional contemporary trials and imaging modalities.

A prior network meta-analysis evaluating thirty two randomized controlled trials and more than twenty two thousand patients found that intravascular imaging guidance was superior to angiography in reducing major adverse cardiovascular events, cardiovascular death, myocardial infarction, stent thrombosis, and target lesion revascularization, with imaging ranking first across most outcomes (66, 67). Our results are highly concordant with these findings, while additionally providing a unified probabilistic comparison across angiography, physiological indices, and intravascular imaging modalities.

Importantly, the present analysis does not demonstrate definitive superiority of intravascular imaging guidance over invasive physiological guidance. There were no statistically significant differences between intravascular ultrasound or optical coherence tomography and fractional flow reserve for the majority of primary and secondary clinical outcomes. Although intravascular imaging modalities ranked numerically higher than instantaneous wave free ratio for certain secondary endpoints, these findings should be interpreted cautiously due to the small number of instantaneous wave free ratio trials and the resulting wide confidence intervals. These differences may therefore reflect imprecision rather than true differences in effectiveness. Notably, fractional flow reserve guided PCI was associated with improved outcomes compared with angiography for cardiac death, underscoring the continued clinical relevance of physiological lesion assessment.

Another meta-analysis further emphasized the clinical value of intravascular imaging guidance, particularly in complex lesions, left main disease, and multivessel disease (4). That analysis demonstrated improved stent expansion, reduced minimal stent area variability, and enhanced procedural optimization with imaging guidance, mechanistic advantages that likely contribute to reductions in ischemic events observed across meta-analyses, including the present study. Optical coherence tomography in particular provides high resolution assessment of stent apposition, edge dissections, and residual thrombus, features that may support improved procedural optimization.

The observed differences among PCI guidance strategies may be partly explained by distinct physiological and procedural mechanisms. Intravascular imaging provides direct, high-resolution assessment of lesion morphology, plaque composition, vessel size, and stent related factors such as expansion, apposition, edge dissections, and residual disease. These procedural insights allow operators to optimize stent sizing, deployment pressure, and post dilation strategies, which are not reliably achievable with angiography alone. Such optimization has been consistently associated with improved minimal stent area, reduced stent under expansion, and lower rates of stent related complications, offering a plausible mechanistic explanation for the superior outcomes of imaging guided PCI compared with angiography observed across randomized trials and meta-analyses.

Physiological indices, including fractional flow reserve and instantaneous wave free ratio, primarily inform lesion selection by identifying ischemia producing stenoses but provide limited guidance for procedural optimization once PCI is undertaken. This distinction may explain why physiological guidance performs favorably compared with angiography for ischemic outcomes, including cardiac death, yet does not consistently differ from intravascular imaging guidance for post PCI clinical endpoints. The numerically lower ranking observed for instantaneous wave free ratio in the present analysis should be interpreted cautiously. Possible explanations include its reliance on resting coronary physiology, which may be influenced by microvascular dysfunction, variable resting flow conditions, and hemodynamic instability, particularly in acute coronary syndrome populations. Additionally, the limited number of randomized trials evaluating instantaneous wave free ratio guided PCI and the resulting wide confidence intervals reduce the precision of effect estimates, raising the possibility that the observed differences reflect statistical imprecision rather than true inferiority. Further randomized studies directly comparing physiological strategies with adequate power are required to clarify these observations.

Taken together, these findings indicate that both invasive physiological guidance and intravascular imaging guidance represent effective alternatives to angiography alone. Current evidence does not support identification of a single advanced guidance strategy that is definitively superior across all clinical outcomes. Physiological indices remain essential for lesion selection and deferral decisions, while intravascular imaging provides complementary information for procedural optimization, including stent sizing, expansion, and detection of edge pathology. The absence of consistent differences between imaging guided and physiology guided strategies likely reflects their complementary roles rather than true equivalence or inferiority.

In contrast to prior meta-analyses evaluating PCI guidance strategies, the present study provides a more comprehensive and contemporary comparative assessment across angiography, physiological indices, and intravascular imaging modalities. Earlier network meta-analyses were limited by smaller sample sizes, fewer optical coherence tomography and instantaneous wave free ratio-based trials, and reliance on older generation stent platforms, which constrained precision and led to cautious conclusions regarding modality performance. By incorporating a substantially larger evidence base of fifty randomized trials and nearly forty thousand patients, including multiple recent large scale contemporary studies, this analysis achieved improved network connectivity, greater statistical power, and more stable treatment rankings.

Importantly, while earlier syntheses emphasized the absence of clear differences among non-angiographic strategies, the expanded dataset in the present analysis allowed clearer discrimination between angiography and advanced guidance approaches, while confirming that differences among physiological and imaging-based strategies remain modest, outcome dependent, and limited by imprecision for some modalities. Despite inherent limitations of trial level network meta-analysis, including heterogeneity in endpoint definitions and variation in how guidance strategies were applied, the consistency of findings across outcomes and sensitivity analyses supports the robustness of these conclusions.

Overall, this network meta-analysis demonstrates that advanced PCI guidance strategies using physiological or intravascular imaging approaches are superior to angiography alone, but does not establish definitive superiority of one non angiographic modality over another. Rather than supporting a single preferred strategy, these findings underscore the importance of selecting PCI guidance based on clinical context, lesion complexity, and procedural objectives. Broader implementation of advanced guidance strategies will require consideration of cost, procedural time, and operator expertise, as previously noted (68). Future randomized trials and individual patient level meta-analyses are warranted to further clarify the optimal integration of physiological and imaging guidance in contemporary PCI practice.

## 5 Limitations

Several limitations should be acknowledged. Although overall statistical heterogeneity was generally low, precision varied across guidance strategies, with wide confidence intervals for modalities supported by fewer randomized trials, particularly instantaneous wave free ratio and, to a lesser extent, optical coherence tomography. The very small number of trials evaluating instantaneous wave free ratio limits the reliability of modality specific comparisons. Clinical and methodological heterogeneity across trials, including differences in patient populations, lesion complexity, stent type and generation, and how guidance strategies were applied for lesion selection vs. procedural optimization, may have influenced results. Subgroup network meta-analyses were not performed because of limited data within individual network nodes and the risk of network fragmentation and ecological bias. As a trial level analysis, the absence of individual patient level data precluded adjustment for patient specific confounders and robust assessment of effect modification. In addition, variation in outcome reporting according to intention to treat or per protocol populations across trials may have contributed to residual variability. Finally, unmeasured confounding related to operator experience and procedural execution could not be addressed.

## 6 Conclusion

This network meta-analysis shows that advanced PCI guidance strategies, including invasive physiological assessment and intravascular imaging, are consistently associated with better clinical outcomes than angiography alone across a broad range of endpoints. Intravascular imaging with IVUS and OCT demonstrated favorable and stable performance across multiple ischemic outcomes, while physiological guidance, particularly FFR, also provided meaningful clinical benefit. However, no single non-angiographic strategy could be identified as definitively superior to the others. These findings support the use of advanced guidance beyond angiography in contemporary PCI and emphasize tailoring guidance strategies to clinical context, lesion complexity, and procedural goals to optimize patient outcomes.

## Data Availability

The original contributions presented in the study are included in the article/Supplementary Material, further inquiries can be directed to the corresponding author/s.
