# Supplementary figures and images for: Comparative effectiveness and outcomes of physiology- and imaging-guided PCI: an evidence synthesis and network meta-analysis of FFR, iFR, OCT, and IVUS
Source: Front Cardiovasc Med. 2026 Mar 20;13:1762634. doi: 10.3389/fcvm.2026.1762634 (PMC13047158; doi:10.3389/fcvm.2026.1762634)

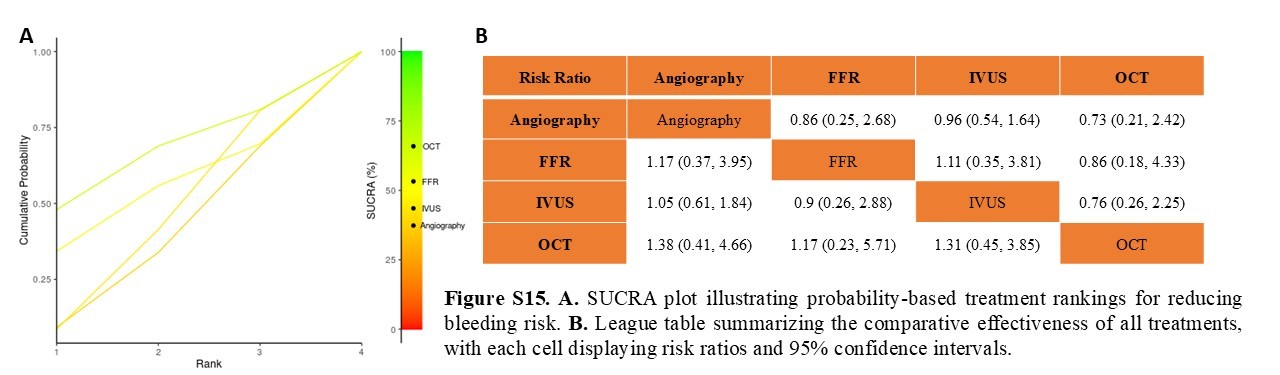

Supplement: Supplementary file 1 [file Image15.jpeg]

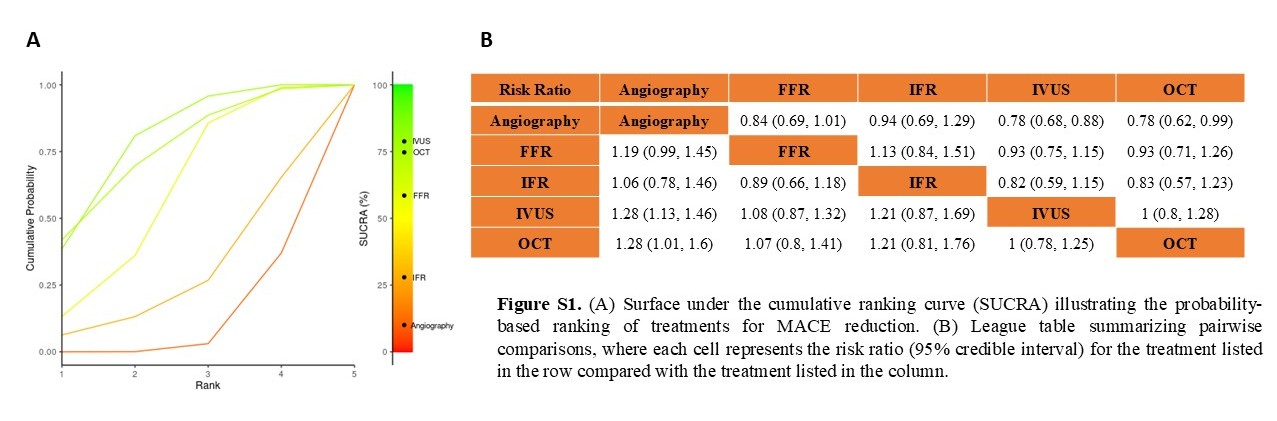

Supplement: Supplementary file 5 [file Image1.jpeg]

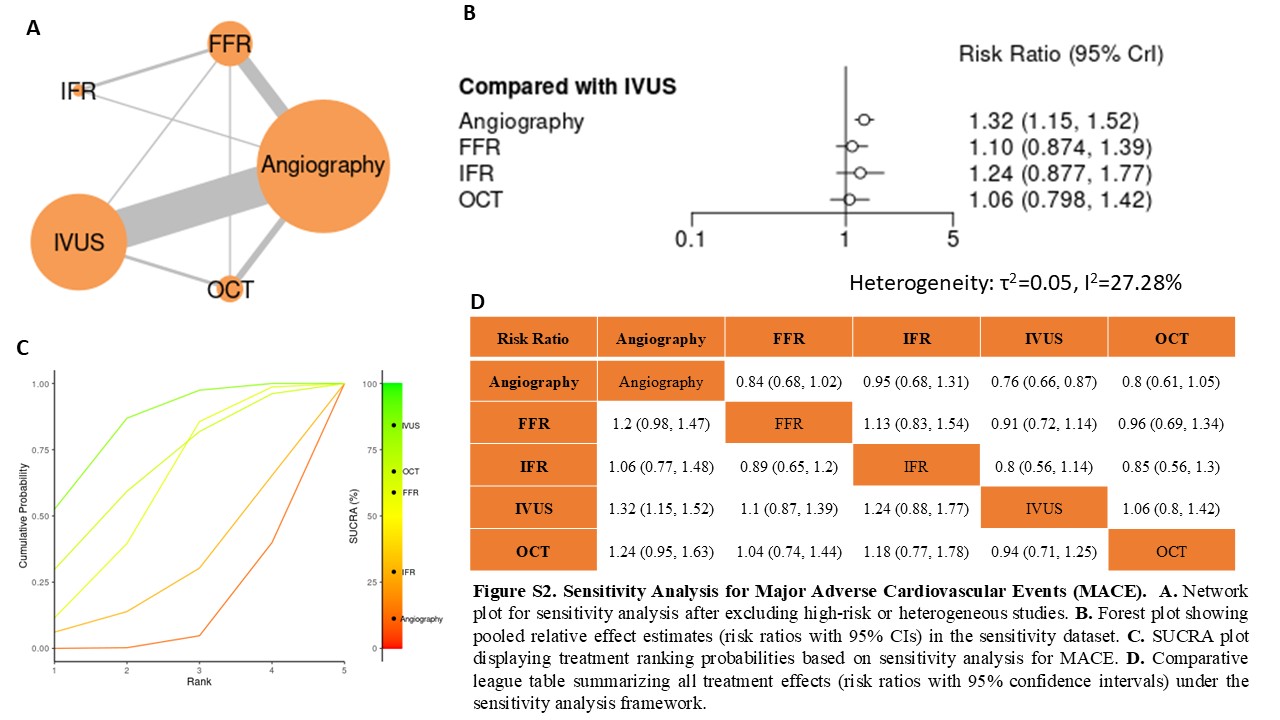

Supplement: Supplementary file 6 [file Image2.jpeg]

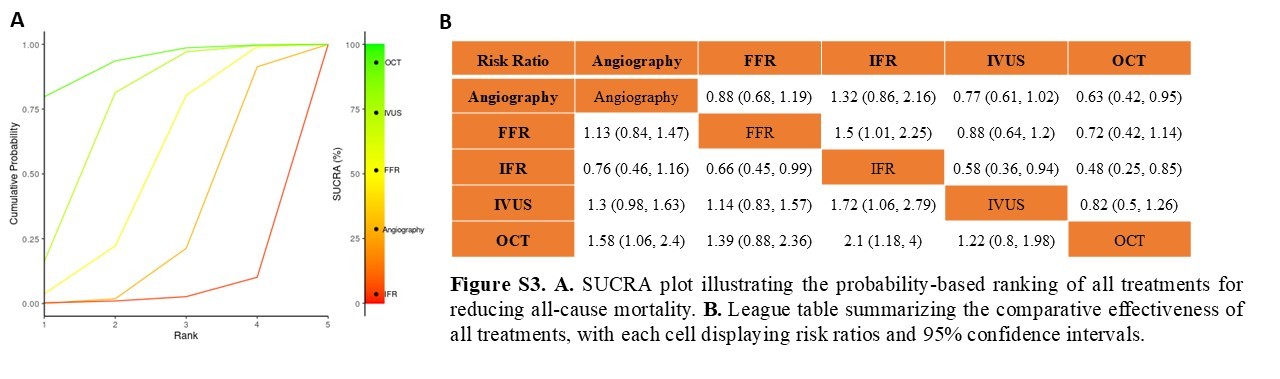

Supplement: Supplementary file 7 [file Image3.jpeg]

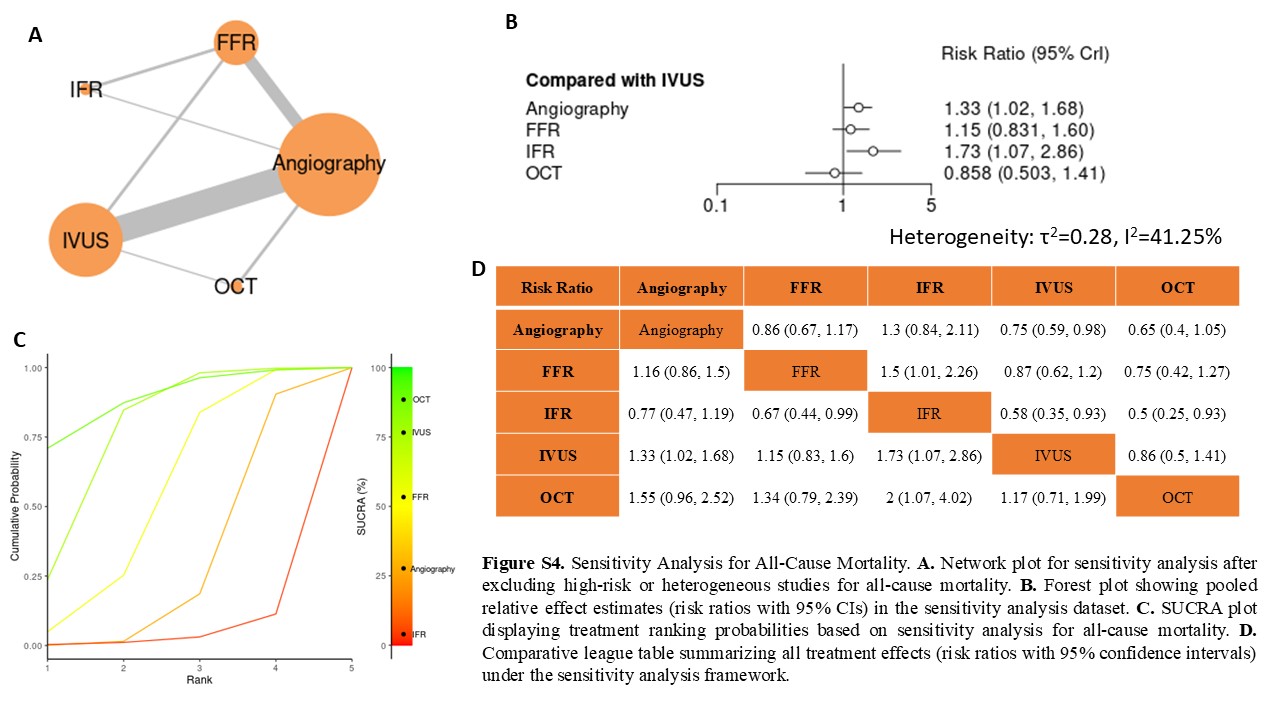

Supplement: Supplementary file 8 [file Image4.jpeg]

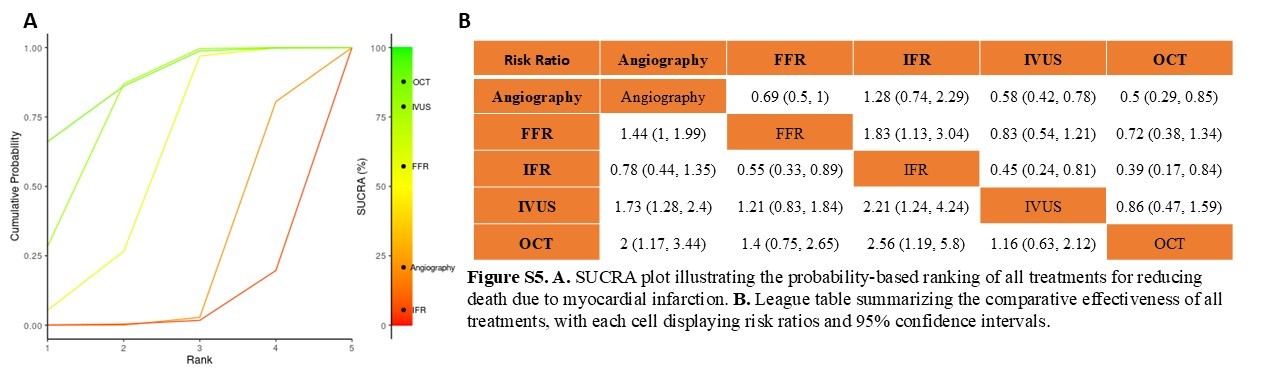

Supplement: Supplementary file 9 [file Image5.jpeg]

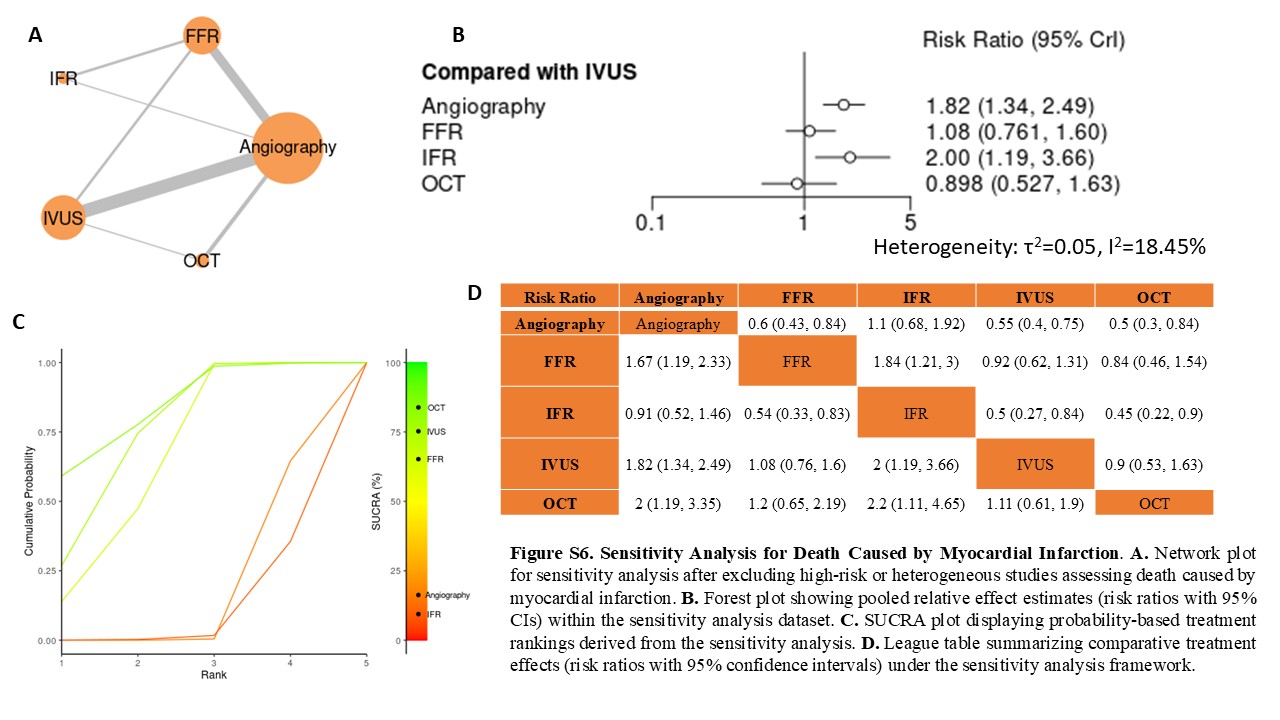

Supplement: Supplementary file 10 [file Image6.jpeg]

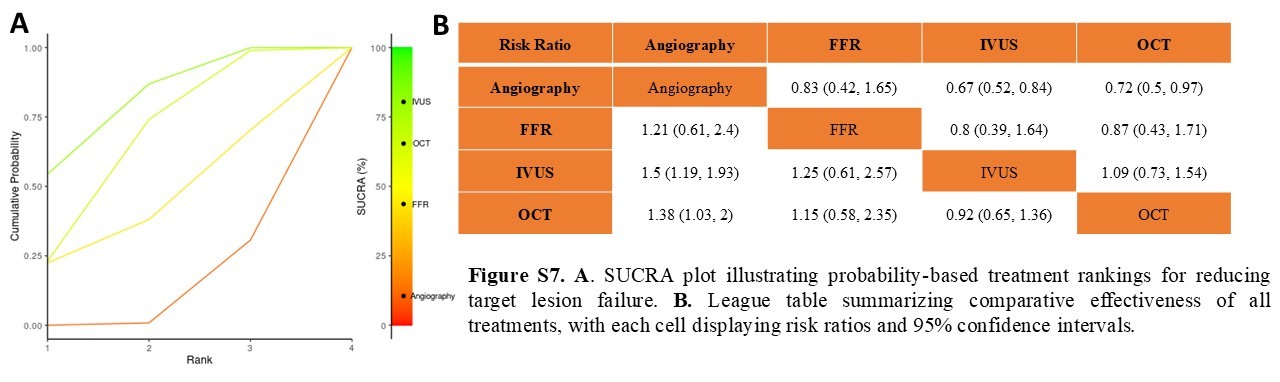

Supplement: Supplementary file 11 [file Image7.jpeg]

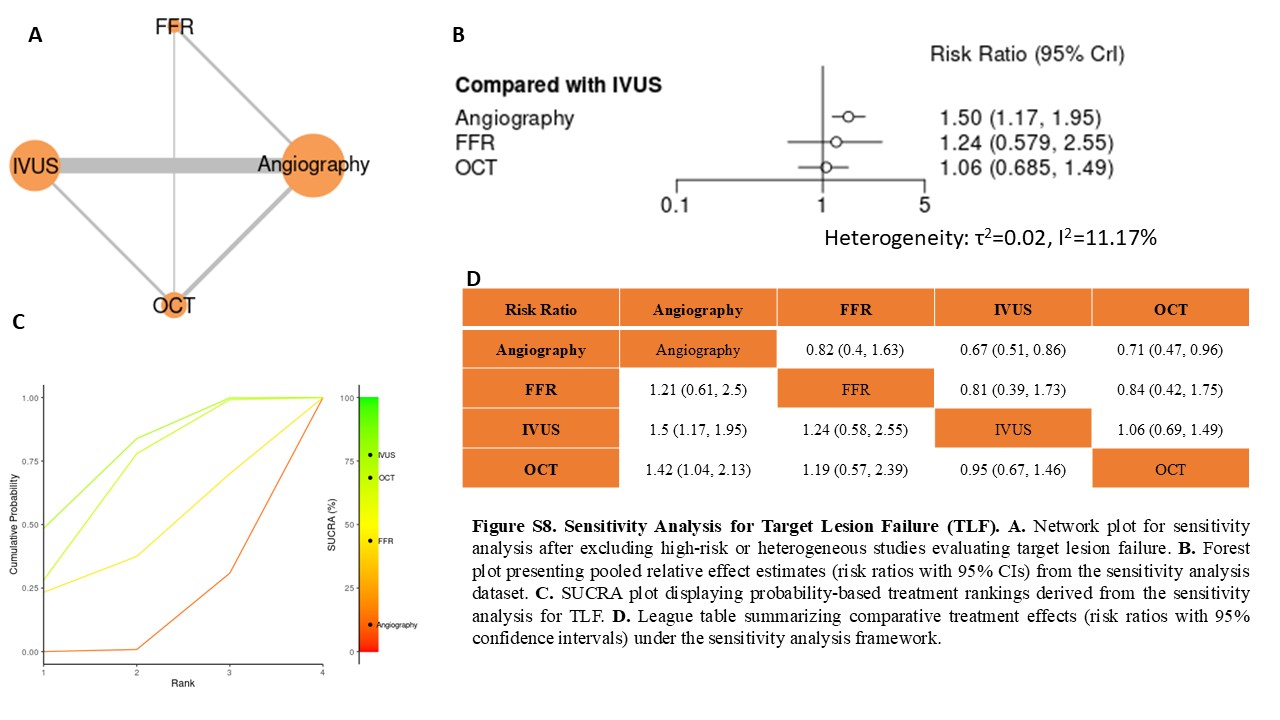

Supplement: Supplementary file 12 [file Image8.jpeg]

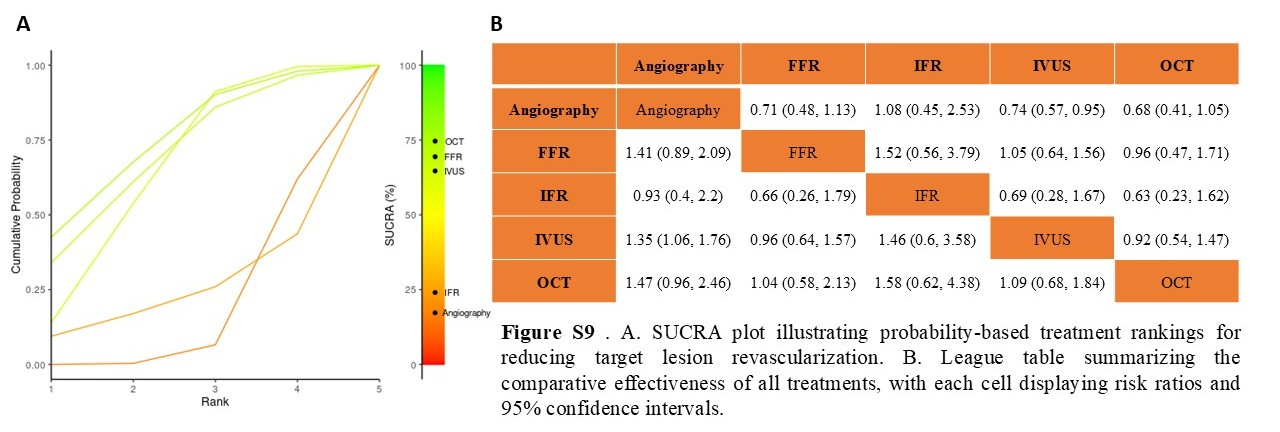

Supplement: Supplementary file 13 [file Image9.jpeg]

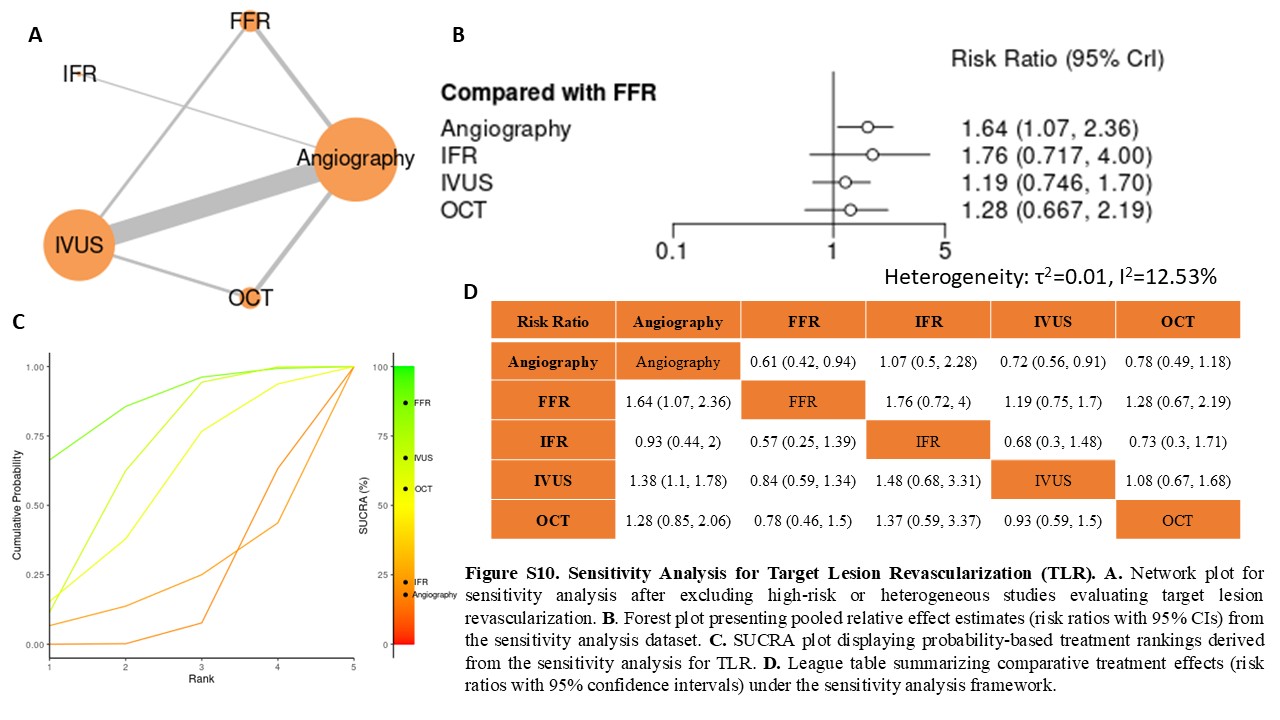

Supplement: Supplementary file 14 [file Image10.jpeg]

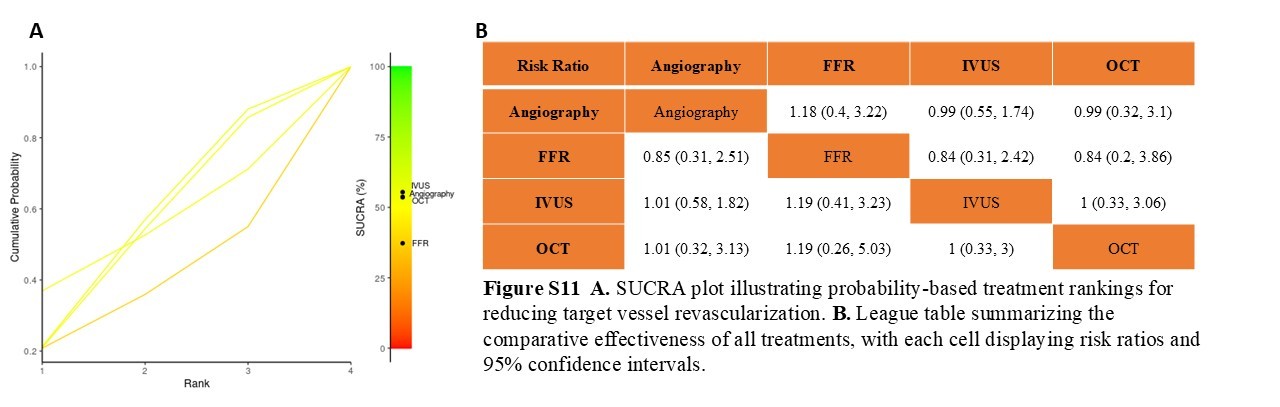

Supplement: Supplementary file 15 [file Image11.jpeg]

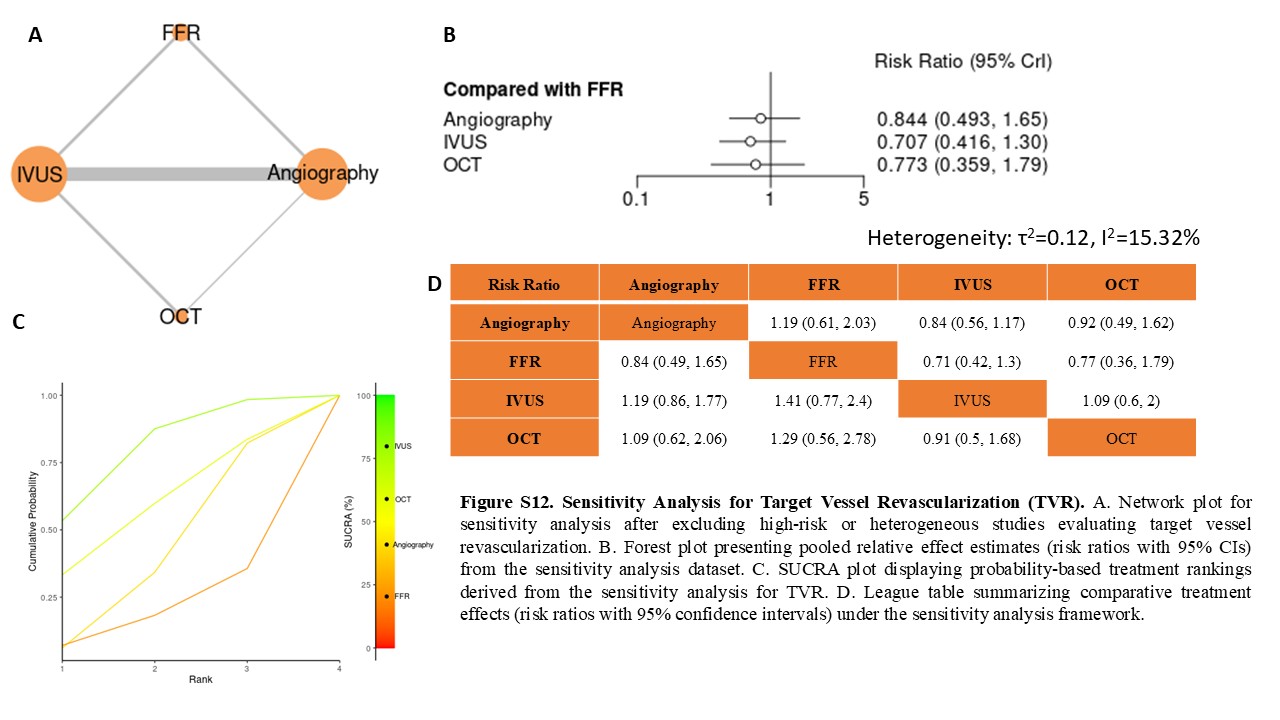

Supplement: Supplementary file 16 [file Image12.jpeg]

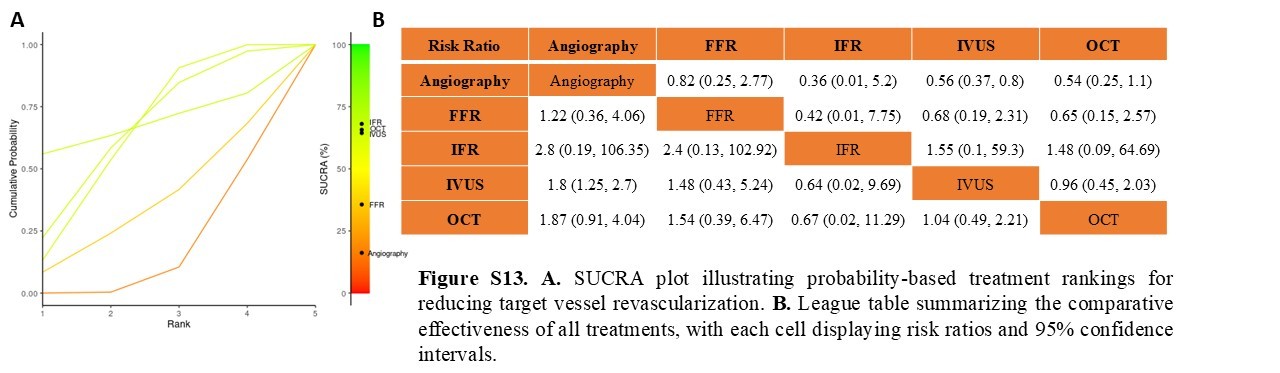

Supplement: Supplementary file 17 [file Image13.jpeg]

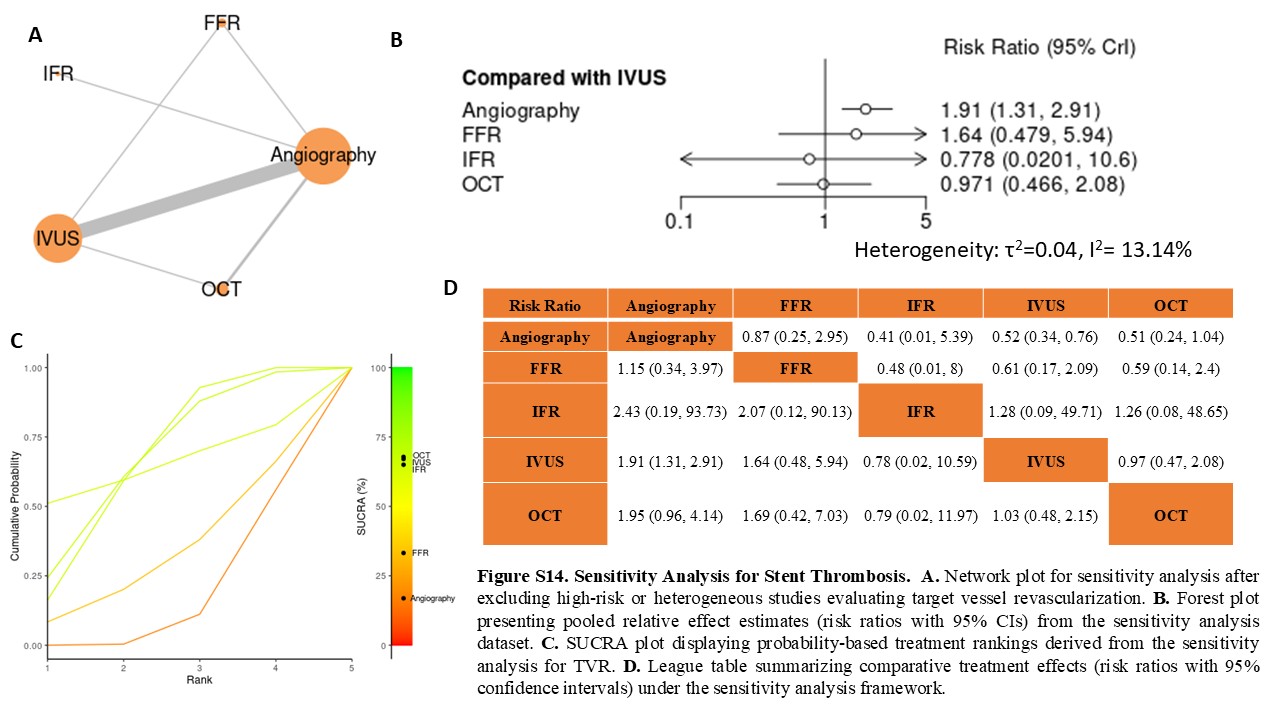

Supplement: Supplementary file 18 [file Image14.jpeg]
