# Supplementary material for: Comparative effectiveness and outcomes of physiology- and imaging-guided PCI: an evidence synthesis and network meta-analysis of FFR, iFR, OCT, and IVUS
Source: Front Cardiovasc Med. 2026 Mar 20;13:1762634. doi: 10.3389/fcvm.2026.1762634 (PMC13047158; doi:10.3389/fcvm.2026.1762634)
Supplement: Supplementary file 2 [file Table1.docx]

Supplementary Table 1. Characteristics of Included Trials and Use of Intravascular/Physiologic Modalities for PCI Guidance and Optimization.

| **Ref No** | **Author Year** | **Modality** | **Decision-making** | **PCI optimization** | **MACE Defintion** |
| --- | --- | --- | --- | --- | --- |
| 16 | Li et. Al. 2024 | IVUS | Yes | Yes | Major Adverse Cardiovascular Events (MACE) were defined as a composite of all-cause death, myocardial infarction, and repeat revascularisation |
| 17 | Yang et. Al. 2025 | FFR | Yes | Yes | a composite of all-cause death, myocardial infarction, and any revascularization occurring after the index procedure. |
| 18 | Stone et. Al. 2025 | IVUS | Yes | Yes | cardiac death, target vessel–related myocardial infarction, or ischemia-driven target vessel revascularization. |
| 19 | Quintella et. Al. 2019 | FFR | Yes | No | MACE (Major Adverse Cardiovascular Events) was defined as a composite of: death (cardiovascular or non-cardiovascular), angina, and target-lesion revascularisation (due to restenosis). |
| 20 | Liu et. Al. 2019 | IVUS | Yes | Yes | MACE (Major Adverse Cardiovascular Events) was defined as a composite of: death (cardiovascular or non-cardiovascular), angina, and target-lesion revascularisation (due to restenosis). |
| 21 | Burzotta et. Al. 2020 | OCT | Yes | Yes | MACE was defined as a composite of: death, myocardial infarction, and target vessel revascularization. |
| 22 | Jakabcin et. Al. 2010 | Angiography | No | Yes | MACE was defined as a composite of: death, myocardial infarction, and target vessel revascularization. |
| 23 | Groenland et. Al. 2025 | IVUS | Yes | Yes | MACE was defined as a composite of: death, myocardial infarction, and target vessel revascularization. |
| 24 | Chamie et. Al. 2021 | OCT | Yes | Yes | MACE was defined as a composite of: death, myocardial infarction, and target vessel revascularization. |
| 25 | Chen et. Al. 2015 | Angiography | Yes | No | MACE was defined as a composite of: death, myocardial infarction, and target vessel revascularization. |
| 26 | Zhang et. Al. 2016 | FFR | Yes | Yes | MACE was defined as a composite of: death, myocardial infarction, and target vessel revascularization. |
| 27 | Wang et. Al. 2015 | IVUS | Yes | Yes | MACE was defined as a composite of: death, myocardial infarction, and target vessel revascularization. |
| 28 | Lee et. Al. 2023 | FFR | Yes | Yes | MACE was defined as a composite of: death, myocardial infarction, and target vessel revascularization. |
| 29 | Ali et. Al. 2025 | OCT | Yes | No | MACE was defined as a composite of: death, myocardial infarction, and target vessel revascularization. |
| 30 | Layland et. Al. 2015 | FFR | Yes | No | MACE was defined as a composite of: death, myocardial infarction, and target vessel revascularization. |
| 31 | Kubo et. Al. 2017 | Angiography | Yes | Yes | MACE was defined as a composite of: death, myocardial infarction, and target vessel revascularization. |
| 32 | Ali et. Al. 2016 | OCT | Yes | Yes | Major adverse cardiovascular events (MACE) were defined as a composite endpoint comprising all-cause mortality, myocardial infarction, and target-vessel revascularization. |
| 33 | Otake et. Al. 2024 | Angiography | Yes | Yes | MACE was defined as a composite of: death, myocardial infarction, and target vessel revascularization. |
| 34 | Frey et. Al. 2000 | IVUS | Yes | Yes | MACE was defined as a composite of: death, myocardial infarction, and target vessel revascularization. |
| 35 | Gaster et. Al. 2003 | IVUS | Yes | Yes | Major adverse cardiovascular events (MACE) were defined as a composite endpoint comprising all-cause mortality, myocardial infarction, and target-vessel revascularization. |
| 36 | Gil et. Al. 2007 | Angiography | Yes | Yes | Major adverse cardiovascular events (MACE) were defined as a composite endpoint comprising all-cause mortality, myocardial infarction, and target-vessel revascularization. |
| 37 | Chieffo et. Al. 2013 | IVUS | Yes | Yes | Major adverse cardiovascular events (MACE) were defined as a composite endpoint comprising all-cause mortality, myocardial infarction, and target-vessel revascularization. |
| 38 | Yoon et. Al. 2013 | IVUS | Yes | Yes | Major adverse cardiovascular events (MACE) were defined as a composite endpoint comprising all-cause mortality, myocardial infarction, and target-vessel revascularization. |
| 39 | Ueki et. Al. 2020 | OCT | Yes | Yes | Major adverse cardiovascular events (MACE) were defined as a composite endpoint comprising all-cause mortality, myocardial infarction, and target-vessel revascularization. |
| 40 | Zhang et. Al. 2018 | IVUS | Yes | Yes | Major adverse cardiovascular events (MACE) were defined as a composite endpoint comprising all-cause mortality, myocardial infarction, and target-vessel revascularization. |
| 41 | Hong et. Al. 2015 | IVUS | Yes | Yes | Major adverse cardiovascular events (MACE) were defined as a composite endpoint comprising all-cause mortality, myocardial infarction, and target-vessel revascularization. |
| 42 | Tan et. Al. 2015 | IVUS | Yes | Yes | Major adverse cardiovascular events (MACE) were defined as a composite endpoint comprising all-cause mortality, myocardial infarction, and target-vessel revascularization. |
| 43 | Stables et. Al. 2022 | FFR | Yes | Yes | Major adverse cardiovascular events (MACE) were defined as a composite endpoint comprising all-cause mortality, myocardial infarction, and target-vessel revascularization. |
| 44 | Tian et. Al. 2015 | IVUS | Yes | Yes | MACE was defined as a composite of: death, myocardial infarction, and target vessel revascularization. |
| 45 | Götberg et. Al. 2017 | IFR | Yes | Yes | MACE was defined as a composite of: death, myocardial infarction, and target vessel revascularization. |
| 46 | Maznyczka et. Al. 2023 | IVUS | Yes | Yes | MACE was defined as a composite of: death, myocardial infarction, and target vessel revascularization. |
| 47 | Russo et. Al. 2009 | IVUS | Yes | Yes | MACE was defined as a composite of: death, myocardial infarction, and target vessel revascularization. |
| 48 | Rioufol et. Al. 2021 | FFR | Yes | Yes | MACE was defined as a composite of: death, myocardial infarction, and target vessel revascularization. |
| 49 | Lee et. Al. 2024 | Angiography | Yes | Yes | Major adverse cardiovascular events (MACE) were defined as a composite endpoint comprising all-cause mortality, myocardial infarction, and target-vessel revascularization. |
| 50 | Park et. Al. 2015 | FFR | Yes | Yes | MACE was defined as a composite of: death, myocardial infarction, and target vessel revascularization. |
| 51 | Oemrawsingh et. Al. 2003 | IVUS | Yes | Yes | MACE was defined as a composite of: death, myocardial infarction, and target vessel revascularization. |
| 52 | Amabile et. Al. 2025 | OCT | Yes | Yes | MACE was defined as a composite of: death, myocardial infarction, and target vessel revascularization. |
| 53 | Mariani et. Al. 2014 | IVUS | Yes | Yes | MACE was defined as a composite of: death, myocardial infarction, and target vessel revascularization. |
| 54 | Holm et. Al. 2023 | OCT | Yes | Yes | Major adverse cardiovascular events (MACE) were defined as a composite endpoint comprising all-cause mortality, myocardial infarction, and target-vessel revascularization. |
| 55 | Ali et. Al. 2023 | OCT | Yes | Yes | Major adverse cardiovascular events (MACE) were defined as a composite endpoint comprising all-cause mortality, myocardial infarction, and target-vessel revascularization. |
| 56 | Puymirat et. Al. 2021 | FFR | Yes | Yes | Major adverse cardiovascular events (MACE) were defined as a composite endpoint comprising all-cause mortality, myocardial infarction, and target-vessel revascularization. |
| 57 | Davies et. Al. 2017 | IFR | Yes | No | Major adverse cardiovascular events (MACE) were defined as a composite endpoint comprising all-cause mortality, myocardial infarction, and target-vessel revascularization. |
| 58 | Tonino et. Al. 2009 | FFR | Yes | Yes | Major adverse cardiovascular events (MACE) were defined as a composite endpoint comprising all-cause mortality, myocardial infarction, and target-vessel revascularization. |
| 59 | Mudra et. Al. 2001 | IVUS | Yes | Yes | MACE was defined as a composite of: death, myocardial infarction, and target vessel revascularization. |
| 60 | Muramatsu et et. Al. 2020 | FFR | Yes | Yes | MACE was defined as a composite of: death, myocardial infarction, and target vessel revascularization. |
| 61 | Vasiljevs et. Al. 2023 | IFR | Yes | Yes | MACE was defined as a composite of: death, myocardial infarction, and target vessel revascularization. |
| 62 | Jia et. Al. 2025 | IVUS | Yes | No | MACE was defined as a composite of: death, myocardial infarction, and target vessel revascularization. |
| 63 | Kim et et. Al. 2015 | IVUS | Yes | Yes | Major adverse cardiovascular events (MACE) were defined as a composite endpoint comprising all-cause mortality, myocardial infarction, and target-vessel revascularization. |
| 64 | Kang et. Al. 2023 | OCT | Yes | No | Major adverse cardiovascular events (MACE) were defined as a composite endpoint comprising all-cause mortality, myocardial infarction, and target-vessel revascularization. |
| 65 | Escaned et. Al. 2024 | IFR | Yes | Yes | Major adverse cardiovascular events (MACE) were defined as a composite endpoint comprising all-cause mortality, myocardial infarction, and target-vessel revascularization. |
