# Supplementary material for: Comparative effectiveness and outcomes of physiology- and imaging-guided PCI: an evidence synthesis and network meta-analysis of FFR, iFR, OCT, and IVUS
Source: Front Cardiovasc Med. 2026 Mar 20;13:1762634. doi: 10.3389/fcvm.2026.1762634 (PMC13047158; doi:10.3389/fcvm.2026.1762634)
Supplement: Supplementary file 3 [file Table2.docx]

Supplementary Table 2. Basic Details of the Study

| **Ref No** | **Author Year** | **Country** | **Total Sample** | **Male** | **Female** | **Age** | **Follow Up** | **Modality** | **Sample** | **Control Modality** | **Sample** | **GRADE** | **Main Finding** |
| --- | --- | --- | --- | --- | --- | --- | --- | --- | --- | --- | --- | --- | --- |
| 16 | Li et. Al. 2024 | China | 3505 | 2584 | 921 | 62 | 12 | IVUS | 1753 | Angiography | 1752 | Moderate | In patients with acute coronary syndromes, IVUS-guided PCI with contemporary DES reduced 1-year target vessel failure (4.0% vs 7.3%; HR 0.55, 95% CI 0.41–0.74) compared with angiography-guided PCI, mainly by lowering target vessel MI and repeat revascularisation, without increasing major bleeding or all-cause mortality. |
| 17 | Yang et. Al. 2025 | China | 1682 | 1187 | 495 | 65.1 | 75.6 | FFR | 838 | IVUS | 844 | Moderate | FFR-guided and IVUS-guided strategies yielded comparable long-term patient-oriented outcomes (death, MI, any revascularization), but FFR guidance had fewer overall target-vessel PCIs at the cost of more late target-vessel revascularizations after initial deferral. |
| 18 | Stone et. Al. 2025 | USA | 2005 | 1464 | 541 | 70 | 12 | IVUS | 1246 | Angiography | 759 | Moderate | Intravascular imaging–guided PCI of severely calcified lesions reduced 1-year target vessel failure, all-cause and cardiac death, and stent thrombosis compared with angiographic guidance alone, without increasing MI or revascularization complications. |
| 19 | Quintella et. Al. 2019 | Brazil | 70 | 47 | 22 | 62 | 18 | FFR | 34 | Angiography | 35 | High | FFR-guided PCI in multivessel disease reduced the number of lesions treated, stents implanted, and target-lesion revascularization, with cost-effectiveness similar to angiography-guided PCI. |
| 20 | Liu et. Al. 2019 | China | 336 | 214 | 122 | 65 | 12 | IVUS | 167 | Angiography | 169 | Moderate | IVUS-guided DES implantation for unprotected left main coronary artery lesions significantly reduced 1-year MACE, driven mainly by lower cardiac mortality, without a clear difference in MI, TVR, TLR, or stent thrombosis compared with angiography-guided PCI. |
| 21 | Burzotta et. Al. 2020 | Italy | 350 | 261 | 89 | 68 | 13 | OCT | 174 | FFR | 176 | Moderate | In patients with angiographically intermediate coronary lesions, OCT-guided strategy reduced the composite of MACE or significant angina at 13 months compared with FFR-guided strategy, but at the cost of more PCI procedures and higher overall treatment costs. |
| 22 | Jakabcin et. Al. 2010 | Czech Republic | 210 | 105 | 105 | 60.2 | 18 | Angiography | 105 | IVUS | 105 | Moderate | In high-risk patients undergoing DES implantation, routine IVUS guidance did *not* reduce 18-month MACE or stent thrombosis compared with careful angiographic guidance alone. |
| 23 | Groenland et. Al. 2025 | Netherlands | 291 | 235 | 56 | 66 | 24 | IVUS | 145 | Angiography | 146 | High | IVUS-guided PCI optimization in patients with low post-PCI FFR worked similarly well in NSTE-ACS and CCS, yielding comparable improvements in FFR and IVUS lumen/stent areas, with no clear difference in 2-year target vessel failure between the clinical presentations. |
| 24 | Chamie et. Al. 2021 | Brazil | 151 | 105 | 45 | 59.92 | 30 | OCT | 51 | IVUS | 50 | Moderate | In 151 patients undergoing PCI, OCT-guided PCI using an EEM-based sizing algorithm achieved stent expansion non-inferior to IVUS and superior to optimized angiography, without increasing periprocedural complications. |
| 25 | Chen et. Al. 2015 | China | 320 | 237 | 83 | 65.4 | 13 | Angiography | 160 | FFR | 160 | Moderate | FFR-guided provisional side-branch stenting did not reduce 1-year MACE compared with angiography-guided provisional stenting in true coronary bifurcation lesions; both strategies yielded identical 18.1% MACE and similar TVR and ST rates. |
| 26 | Zhang et. Al. 2016 | China | 220 | 153 | 67 | 70 | 12 | FFR | 110 | Angiography | 110 | Moderate | FFR-guided management in elderly Chinese NSTEMI patients reduced the need for PCI (more patients treated with medical therapy alone) while achieving similar 1-year clinical outcomes compared with angiography-guided therapy. |
| 27 | Wang et. Al. 2015 | China | 80 | 51 | 29 | 56.4 | 12 | IVUS | 38 | Angiography | 42 | Moderate | In STEMI patients with high thrombus burden and 50–75% residual stenosis after aspiration, IVUS-guided strategy (allowing conservative management in low-risk lesions) significantly reduced the number of stents implanted without increasing 12-month MACE or impairing LV function compared with routine angiography-guided stenting. |
| 28 | Lee et. Al. 2023 | Korea | 562 | 474 | 88 | 63.3 | 42 | FFR | 284 | Angiography | 278 | Moderate | In AMI patients with multivessel disease, FFR-guided selective PCI of non-culprit lesions reduced the composite of death, MI, or repeat revascularization (7.4% vs 19.7%) and used fewer stents and less contrast than routine angiography-guided PCI. |
| 29 | Ali et. Al. 2025 | USA | 1082 | 830 | 252 | 67.3 | 24 | OCT | 544 | Angiography | 538 | Moderate | In patients with angiographically moderate or severe calcified coronary lesions, OCT-guided PCI reduced 2-year target-vessel failure and serious MACE compared with angiography-guided PCI, mainly by achieving better stent expansion and fewer peri-procedural complications. |
| 30 | Layland et. Al. 2015 | United Kingdom | 350 | 260 | 90 | 62 | 12 | FFR | 176 | Angiography | 174 | High | In NSTEMI patients, FFR-guided management reduced the need for coronary revascularization compared with angiography-guided care, without significant differences in 12-month MACE or quality of life. |
| 31 | Kubo et. Al. 2017 | Japan | 829 | 637 | 180 | 69 | 12 | Angiography | 414 | IVUS | 415 | Moderate | In patients undergoing PCI with second-generation DES, OFDI-guided PCI was non-inferior to IVUS-guided PCI at 12 months, with very low rates of target vessel failure and angiographic restenosis in both groups. |
| 32 | Ali et. Al. 2016 | Multinational | 450 | 323 | 127 | 66 | 12 | OCT | 158 | IVUS | 146 | High | In ILUMIEN III, OCT-guided PCI achieved similar 12-month TLF and MACE rates to IVUS- and angiography-guided PCI, despite better stent expansion and fewer untreated dissections/malappositions with OCT guidance. |
| 33 | Otake et. Al. 2024 | Japan | 134 | 68 | 66 | 16 | 12 | Angiography | 68 | IVUS | 66 | Moderate | OFDI-guided PCI in ACS was non-inferior to IVUS-guided PCI for 8-month in-stent minimum lumen area and showed similar 12-month clinical outcomes, while reducing proximal stent-edge dissection and irregular tissue protrusion and achieving better post-PCI coronary flow. |
| 34 | Frey et. Al. 2000 | Germany | 269 | 212 | 57 | 61.2 | 24 | IVUS | 121 | Angiography | 148 | Moderate | ICUS-guided provisional stenting improved acute angiographic results and significantly reduced 2-year clinically driven target lesion revascularization compared with standard angiographic guidance, despite similar 6-month angiographic minimal lumen diameter. |
| 35 | Gaster et. Al. 2003 | Denmark | 108 | 108 | 0 | 57 | 2.5 | IVUS | 54 | Angiography | 54 | Moderate | IVUS-guided PCI in elective male stable-angina patients reduced long-term MACE and produced substantially lower cumulative costs than angiography-guided PCI. |
| 36 | Gil et. Al. 2007 | Poland | 259 | 70 | 189 | 54 | 6 | Angiography | 80 | IVUS | 83 | Moderate | IVUS-guided direct stenting achieved larger acute lumen gain and significantly lower 6-month MACE and restenosis than angiography-guided direct stenting or IVUS-guided balloon angioplasty in stable angina patients undergoing PCI. |
| 37 | Chieffo et. Al. 2013 | Italy | 284 | 226 | 58 | 63.9 | 24 | IVUS | 142 | Angiography | 142 | Moderate | In patients with complex coronary lesions treated with DES, IVUS-guided stent implantation produced a significantly larger final in-lesion minimal lumen diameter than angiography-guided implantation, but did not reduce clinical MACE up to 24 months. |
| 38 | Yoon et. Al. 2013 | South Korea | 1574 | 1020 | 554 | 61.2 | 12 | IVUS | 662 | Angiography | 912 | Moderate | In patients with relatively simple short-length coronary lesions treated with DESs, routine IVUS-guided PCI did *not* improve 1-year clinical outcomes (MACE, death, MI, TVR) compared with angiography-guided PCI. |
| 39 | Ueki et. Al. 2020 | Switzerland | 38 | 30 | 8 | 63.3 | 12 | OCT | 19 | Angiography | 19 | Moderate | OCT-guided BVS implantation did not improve 6-month in-scaffold minimum lumen area compared with angiography guidance, but achieved better scaffold expansion and fewer malapposed struts without clear differences in 1-year clinical outcomes. |
| 40 | Zhang et. Al. 2018 | China | 1448 | 1065 | 383 | 65.2 | 12 | IVUS | 724 | Angiography | 724 | Moderate | IVUS-guided DES implantation significantly reduced 1-year target-vessel failure compared with angiography guidance in all-comer PCI patients, mainly via fewer repeat revascularizations, without increasing kidney injury. |
| 41 | Hong et. Al. 2015 | Korea | 1400 | 964 | 436 | 64 | 12 | IVUS | 700 | Angiography | 700 | Moderate | In patients with long coronary lesions treated with everolimus-eluting stents, IVUS-guided implantation reduced 1-year MACE (2.9% vs 5.8%), mainly by lowering ischemia-driven target lesion revascularization, compared with angiography-guided PCI. |
| 42 | Tan et. Al. 2015 | China | 123 | 81 | 42 | 76.15 | 23.26 | IVUS | 61 | Angiography | 62 | Low | IVUS-guided PCI reduced 2-year MACE (13.1% vs 29.3%) driven mainly by fewer TLRs compared with angiography-guided PCI in elderly patients with unprotected left main disease. |
| 43 | Stables et. Al. 2022 | United Kingdom | 1100 | 829 | 271 | 64.3 | 12 | FFR | 548 | Angiography | 552 | Moderate | Systematic FFR assessment of all major coronary vessels at diagnostic angiography (Angio+FFR) was cost-neutral and produced no improvement in 1-year quality-of-life or reduction in clinical events compared with angiography alone. |
| 44 | Tian et. Al. 2015 | China | 230 | 115 | 115 | 63.5 | 24 | IVUS | 115 | Angiography | 115 | Moderate | IVUS-guided CTO stenting reduced in-stent late lumen loss and was associated with lower rates of stent thrombosis and fewer true-lumen restenoses compared with angiography guidance, although 2-year MACE rates were similar. |
| 45 | Götberg et. Al. 2017 | Sweden | 2037 | 1522 | 515 | 67.6 | 12 | IFR | 1019 | Angiography | 1018 | Moderate | An iFR-guided revascularization strategy was noninferior to FFR guidance for the 12-month composite of death, nonfatal MI, or unplanned revascularization (6.7% vs 6.1%; noninferiority met) and caused far less chest discomfort during assessment. |
| 46 | Maznyczka et. Al. 2023 | United Kingdom | 455 | 351 | 104 | 71.2 | 12 | IVUS | 229 | Angiography | 226 | Moderate | Intracoronary imaging did not change 1-year outcomes overall, but absence of imaging worsened outcomes in patients undergoing a dual-stent strategy. |
| 47 | Russo et. Al. 2009 | United States | 800 | 564 | 236 | 62 | 12 | IVUS | 394 | Angiography | 406 | Moderate | IVUS-directed bare-metal stent placement produced larger final stent dimensions and—while the overall 12-month TLR difference did not reach statistical significance for the full cohort—IVUS significantly reduced 12-month TLR in prespecified vessel-size and high-grade stenosis subgroups (notably vessels ≥2.5 mm) without increasing complications. |
| 48 | Rioufol et. Al. 2021 | France | 927 | 778 | 149 | 65 | 24 | FFR | 460 | Angiography | 467 | Moderate | An FFR-guided strategy in multivessel CAD significantly increased the proportion of patients managed medically and reduced revascularization, but it did not reduce the 1-year rate of MACCE compared with angiography-guided management. |
| 49 | Lee et. Al. 2024 | Korea | 1528 | 1196 | 332 | 64.6 | 12 | Angiography | 763 | IVUS | 765 | Moderate | QCA-guided PCI produced similar 12-month target-lesion failure (3.81% vs 3.80%) and similar post-PCI minimum lumen diameter to IVUS-guided PCI, suggesting a standardized QCA algorithm may be an acceptable alternative to IVUS when IVUS is unavailable. |
| 50 | Park et. Al. 2015 | Korea | 229 | 170 | 59 | 62 | 60 | FFR | 114 | Angiography | 115 | Moderate | In patients with angiographically intermediate coronary stenosis, an FFR-guided strategy produced comparable 5-year clinical outcomes to routine DES implantation while avoiding DES implantation in many patients (≈75% were FFR≥0.75 and deferred). |
| 51 | Oemrawsingh et. Al. 2003 | Netherlands | 150 | 108 | 42 | 61 | 12 | IVUS | 74 | Angiography | 76 | Moderate | IVUS-guided stenting of long coronary lesions produced larger 6-month minimal lumen diameters and significantly lower rates of restenosis, TLR, and combined cardiac events compared with angiography-guided stenting |
| 52 | Amabile et. Al. 2025 | France | 143 | 109 | 25 | 73 | 1 | OCT | 65 | Angiography | 69 | Moderate | An OCT-guided PCI strategy using a pre-defined algorithm achieved significantly larger final minimal stent area and better stent expansion than angiography-guided PCI for moderate-to-severe calcified lesions, without excess short-term safety events. |
| 53 | Mariani et. Al. 2014 | Brazil | 83 | 49 | 34 | 67 | 4 | IVUS | 41 | Angiography | 42 | Moderate | IVUS-guided PCI markedly and safely reduced the total volume of iodine contrast used (median 20.0 ml vs 64.5 ml; p<0.001) compared with angiography-guided PCI, with no excess clinical adverse events. |
| 54 | Holm et. Al. 2023 | Denmark | 1201 | 948 | 253 | 66.3 | 24 | OCT | 600 | Angiography | 601 | High | At 2 years, OCT-guided PCI for complex coronary bifurcation lesions reduced the incidence of MACE (10.1% vs 14.1%; HR 0.70, 95% CI 0.50–0.98; P=0.035) compared with angiography-guided PCI. |
| 55 | Ali et. Al. 2023 | Global | 2487 | 1924 | 563 | 65.6 | 24 | OCT | 1233 | Angiography | 1254 | High | OCT-guided PCI produced a larger minimum stent area than angiography-guided PCI, but there was no significant difference in target-vessel failure at 2 years; stent thrombosis was lower with OCT. |
| 56 | Puymirat et. Al. 2021 | France | 1171 | 966 | 197 | 62.2 | 12 | FFR | 586 | Angiography | 577 | High | In patients with STEMI and multivessel disease, an FFR-guided strategy for complete revascularization did not significantly reduce the 1-year composite of death, nonfatal MI, or urgent revascularization compared with angiography-guided revascularization (5.5% vs 4.2%; HR 1.32, P=0.31). |
| 57 | Davies et. Al. 2017 | Global | 2492 | 1891 | 601 | 65.5 | 12 | IFR | 1242 | FFR | 1250 | High | iFR-guided coronary revascularization was noninferior to FFR-guided revascularization for 1-year major adverse cardiac events, with fewer procedural symptoms and shorter procedure time in the iFR group. |
| 58 | Tonino et. Al. 2009 | United States | 1005 | 744 | 261 | 64.6 | 12 | FFR | 509 | Angiography | 496 | High | Routine measurement of fractional flow reserve (FFR) to guide PCI in multivessel disease reduced the 1-year rate of the composite of death, nonfatal MI, and repeat revascularization (13.2% FFR vs 18.3% angiography; P = 0.02) |
| 59 | Mudra et. Al. 2001 | Germany | 550 | 426 | 124 | 60.1 | 12 | IVUS | 273 | Angiography | 277 | Moderate | Use of intravascular ultrasound guidance during coronary stent implantation did not reduce 6-month angiographic restenosis or 12-month major adverse cardiac events compared with angiography-guided stent implantation. |
| 60 | Muramatsu et et. Al. 2020 | Japan | 109 | 85 | 24 | 70 | 36 | FFR | 54 | IVUS | 55 | Moderate | OFDI-guided PCI was non-inferior to IVUS-guided PCI for in-segment minimum lumen area at 8 months, with similar neointimal healing and comparable device-oriented clinical outcomes up to 3 years. (Abstract / Conclusions). |
| 61 | Vasiljevs et. Al. 2023 | Latvia | 17 | 10 | 7 | 64 | 3 | IFR | 9 | Angiography | 8 | Low | *One-stage iFR-guided virtual planning complete revascularization is feasible in multivessel ACS, requires fewer PCI attempts and—despite longer index procedure and higher contrast use—showed no increase in PCI-related MACE at 3-month follow-up compared with staged angiography-guided PCI.* |
| 62 | Jia et. Al. 2025 | China | 117 | 100 | 17 | 58.4 | 12 | IVUS | 56 | Angiography | 61 | Low | *IVUS-guided stent implantation (IGSI) for stenosis proximal to myocardial bridge produced more accurate stent positioning, larger lumen and less restenosis and resulted in lower 12-month MACE (5.4% vs 18.0%) and fewer TLR events (0% vs 8.2%) compared with angiography-guided stenting (AGSI).* |
| 63 | Kim et et. Al. 2015 | South Korea | 402 | 324 | 78 | 11.1 | 12 | IVUS | 201 | Angiography | 201 | Moderate | IVUS-guided CTO PCI significantly reduced 12-month MACE compared with angiography-guided PCI. |
| 64 | Kang et. Al. 2023 | South Korea | 2008 | 1575 | 433 | 64.7 | 24 | OCT | 1005 | IVUS | 1003 | Moderate | OCT-guided PCI was noninferior to IVUS-guided PCI for the 1-year composite of cardiac death, target-vessel MI, or ischemia-driven target-vessel revascularization (2.5% vs 3.1%; noninferiority P<0.001). |
| 65 | Escaned et. Al. 2024 | Spain | 2492 | 1891 | 601 | 65.5 | 60 | IFR | 1242 | FFR | 1250 | High | At 5 years, an iFR-based strategy produced similar MACE rates to an FFR-based strategy (21.1% vs 18.4%; HR 1.18; P = .06), but all-cause and cardiovascular mortality were higher in the iFR arm. |
