# Supplementary material for: Comparative effectiveness and outcomes of physiology- and imaging-guided PCI: an evidence synthesis and network meta-analysis of FFR, iFR, OCT, and IVUS
Source: Front Cardiovasc Med. 2026 Mar 20;13:1762634. doi: 10.3389/fcvm.2026.1762634 (PMC13047158; doi:10.3389/fcvm.2026.1762634)
Supplement: Supplementary file 4 [file Table3.docx]

| Supplementary Table 3. Baseline values from the studies and Characteristics. | | | | | | | | | | |
| --- | --- | --- | --- | --- | --- | --- | --- | --- | --- | --- |
| 3-A . Demographics | | | | | | | | | | |
| Author / year | Body mass index | | Diabetes mellitus | | Hypertension | | Hyperlipidemia | | Smoking | |
|  | Modality | Control Modality | Modality | Control Modality | Modality | Control Modality | Modality | Control Modality | Modality | Control Modality |
| Li et. Al. 2024 | N/A | N/A | 554 | 551 | 1103 | 1089 | 1187 | 1222 | 499 | 487 |
| Yang et. Al. 2025 | 24.7 ± 3.3 | 24.7 ± 3.3 | 272 | 282 | 577 | 570 | 667 | 655 | 166 | 155 |
| Stone et. Al. 2025 | 29.1 ± 3.3 | 29.1 ± 3.3 | 563 | 345 | 1117 | 693 | 1102 | 653 | 732 | 466 |
| Quintella et. Al. 2019 | N/A | N/A | 12 | 12 | 25 | 26 | 24 | 26 | 10 | 9 |
| Liu et. Al. 2019 | 23.8 ± 3.8 | 24.1 ± 2.9 | 56 | 52 | 116 | 122 | 63 | 64 | 62 | 60 |
| Burzotta et. Al. 2020 | 27 ± 10 | 27 ± 5 | 61 | 63 | 148 | 151 | 120 | 130 | 70 | 66 |
| Jakabcin et. Al. 2010 | N/A | N/A | 47 | 44 | 75 | 70 | 69 | 66 | 37 | 42 |
| Groenland et. Al. 2025 | N/A | N/A | 7 | 17 | 101 | 107 | 37 | 39 | 63 | 50 |
| Chamie et. Al. 2021 | N/A | N/A | 17 | 20 | 46 | 42 | 36 | 30 | 17 | 14 |
| Chen et. Al. 2015 | N/A | N/A | 43 | 48 | 106 | 116 | 32 | 27 | 64 | 66 |
| Zhang et. Al. 2016 | N/A | N/A | 40 | 36 | 81 | 83 | 90 | 93 | 29 | 31 |
| Wang et. Al. 2015 | N/A | N/A | 8 | 5 | 15 | 10 | 10 | 10 | 19 | 25 |
| Lee et. Al. 2023 | 24.8 ± 3.2 | 24.9 ± 3.4 | 97 | 86 | 151 | 152 | 121 | 107 | 91 | 105 |
| Ali et. Al. 2025 | 28.7 ± 5.4 | 28.7 ± 5.5 | 226 | 213 | 393 | 411 | 353 | 368 | 91 | 83 |
| Layland et. Al. 2015 | N/A | N/A | 26 | 26 | 78 | 81 | 71 | 56 | 72 | 71 |
| Kubo et. Al. 2017 | N/A | N/A | 169 | 165 | 315 | 299 | 316 | 321 | 70 | 61 |
| Ali et. Al. 2016 | 28.1 | 28 | 52 | 55 | 124 | 113 | 115 | 107 | 28 | 19 |
| Otake et. Al. 2024 | N/A | N/A | 25 | 23 | 47 | 37 | 44 | 40 | 9 | 5 |
| Frey et. Al. 2000 | N/A | N/A | 19 | 24 | 77 | 82 | 105 | 129 | 56 | 66 |
| Gaster et. Al. 2003 | N/A | N/A | 6 | 2 | 13 | 11 | 50 | 52 | 8 | 16 |
| Gil et. Al. 2007 | N/A | N/A | 9 | 8 | 32 | 39 | 42 | 39 | 32 | 37 |
| Chieffo et. Al. 2013 | N/A | N/A | 34 | 38 | 100 | 95 | 100 | 109 | 49 | 44 |
| Yoon et. Al. 2013 | N/A | N/A | 184 | 270 | 397 | 570 | N/A | N/A | 167 | 236 |
| Ueki et. Al. 2020 | 27.7 ± 4.1 | 28.2 ± 3.7 | 4 | 4 | 7 | 11 | 13 | 12 | 7 | 6 |
| Zhang et. Al. 2018 | 25.3 | 25.3 | 217 | 226 | 512 | 521 | 389 | 400 | 253 | 228 |
| Hong et. Al. 2015 | 24.6 ± 3.0 | 24.8 ± 3.1 | 250 | 256 | 454 | 444 | 471 | 458 | 155 | 181 |
| Tan et. Al. 2015 | N/A | N/A | 21 | 18 | 25 | 29 | N/A | N/A | 27 | 29 |
| Stables et. Al. 2022 | 29.1 ± 5.2 | 29.1 ± 5.3 | 113 | 97 | 315 | 294 | 315 | 317 | 316 | 356 |
| Tian et. Al. 2015 | N/A | N/A | N/A | N/A | N/A | N/A | N/A | N/A | N/A | N/A |
| Götberg et. Al. 2017 | 27.6 ± 4.3 | 27.6 ± 4.3 | 232 | 213 | 730 | 710 | 733 | 704 | 159 | 167 |
| Maznyczka et. Al. 2023 | 28.8 ± 6.3 | 28.3 ± 4.8 | 44 | 82 | 145 | 221 | 134 | 187 | 30 | 36 |
| Russo et. Al. 2009 | N/A | N/A | 59 | 69 | 181 | 183 | 158 | 178 | N/A | N/A |
| Rioufol et. Al. 2021 | **28 ± 5** | 27 ± 5 | 143 | 147 | 265 | 283 | 275 | 286 | 109 | 118 |
| Lee et. Al. 2024 | 24.8 ± 3.1 | 24.9 ± 2.7 | 257 | 237 | 480 | 488 | 655 | 649 | 203 | 178 |
| Park et. Al. 2015 | N/A | N/A | 30 | 39 | 73 | 65 | 80 | 78 | 30 | 38 |
| Oemrawsingh et. Al. 2003 | N/A | N/A | 12 | 16 | 20 | 23 | 45 | 47 | 30 | 40 |
| Amabile et. Al. 2025 | 26.8 | 26 | 24 | 27 | 46 | 43 | 56 | 61 | 8 | 10 |
| Mariani et. Al. 2014 | N/A | N/A | 30 | 34 | 40 | 42 | N/A | N/A | 2 | 3 |
| Holm et. Al. 2023 | 28.0 ± 4.6 | 28.2 ± 4.9 | 103 | 97 | 422 | 448 | 456 | 471 | 77 | 85 |
| Ali et. Al. 2023 | 28.7 ± 5.3 | 28.8 ± 5.5 | 523 | 521 | 880 | 928 | 808 | 860 | 242 | 303 |
| Puymirat et. Al. 2021 | 26.7 | 26.6 | 107 | 82 | 253 | 262 | 232 | 237 | 235 | 210 |
| Davies et. Al. 2017 | 27.8 ± 5.0 | **27.5 ± 5.0** | 382 | 376 | 873 | 884 | 794 | 792 | 461 | 443 |
| Tonino et. Al. 2009 | N/A | N/A | 123 | 125 | 312 | 327 | 366 | 362 | 138 | 156 |
| Mudra et. Al. 2001 | N/A | N/A | 46 | 47 | 131 | 144 | 167 | 186 | 188 | 183 |
| Muramatsu et et. Al. 2020 | N/A | N/A | 27 | 24 | 34 | 39 | 43 | 36 | 22 | 12 |
| Vasiljevs et. Al. 2023 | 26.1 ± 4.8 | 29.3 ± 4.1 | N/A | 3 | 5 | 7 | N/A | N/A | 5 | 4 |
| Jia et. Al. 2025 | 24.56 ± 3.07 | 24.97 ± 3.05 | 14 | 19 | 38 | 46 | N/A | N/A | 26 | 33 |
| Kim et et. Al. 2015 | N/A | N/A | 70 | 68 | 126 | 128 | N/A | N/A | 71 | 69 |
| Kang et. Al. 2023 | 24.9 ± 3.2 | 25.0 ± 3.1 | 325 | 345 | 647 | 639 | 840 | 841 | 217 | 189 |
| Escaned et. Al. 2024 | N/A | N/A | N/A | N/A | N/A | N/A | N/A | N/A | N/A | N/A |
| Amabile et. Al. 2025 | 26.8 | 26 | 24 | 27 | 46 | 43 | 56 | 61 | 8 | 10 |
| **3-B. Cardiovascular History** | | | | | | | | | | |
| Author / year | Myocardial Infarction | | PCI | | CABG | | Heart Failure | | CKD | |
|  | Modality | Control Modality | Modality | Control Modality | Modality | Control Modality | Modality | Control Modality | Modality | Control Modality |
| Li et. Al. 2024 | 152 | 154 | 179 | 179 | 4 | 4 | 111 | 106 | 132 | 127 |
| Yang et. Al. 2025 | 56 | 39 | 165 | 163 | N/A | N/A | N/A | N/A | 143 | 147 |
| Stone et. Al. 2025 | 333 | 201 | 550 | 356 | 118 | 83 | N/A | N/A | 265 | 214 |
| Quintella et. Al. 2019 | 8 | 7 | N/A | N/A | N/A | N/A | N/A | N/A | N/A | N/A |
| Liu et. Al. 2019 | 29 | 24 | 33 | 28 | 2 | 2 | 31 | 33 | N/A | N/A |
| Burzotta et. Al. 2020 | 33 | 52 | 73 | 76 | 4 | 5 | N/A | N/A | 32 | 30 |
| Jakabcin et. Al. 2010 | 34 | 39 | 15 | 18 | 10 | 5 | N/A | N/A | N/A | N/A |
| Groenland et. Al. 2025 | 36 | 32 | 47 | 45 | 7 | 5 | N/A | N/A | N/A | N/A |
| Chamie et. Al. 2021 | 9 | 10 | N/A | N/A | N/A | N/A | N/A | N/A | N/A | N/A |
| Chen et. Al. 2015 | 19 | 12 | 19 | 26 | 1 | 1 | N/A | N/A | 32 | 28 |
| Zhang et. Al. 2016 | 24 | 23 | N/A | N/A | N/A | N/A | 2 | 1 | N/A | N/A |
| Wang et. Al. 2015 | N/A | N/A | N/A | N/A | N/A | N/A | N/A | N/A | N/A | N/A |
| Lee et. Al. 2023 | 7 | 7 | 17 | 20 | N/A | N/A | N/A | N/A | N/A | N/A |
| Ali et. Al. 2025 | 106 | 118 | 71 | 76 | 32 | 26 | N/A | N/A | N/A | N/A |
| Layland et. Al. 2015 | 55 | 47 | 49 | 56 | 22 | 24 | N/A | N/A | N/A | N/A |
| Kubo et. Al. 2017 | 70 | 61 | 140 | 140 | 7 | 9 | N/A | N/A | N/A | N/A |
| Ali et. Al. 2016 | 35 | 29 | 11 | 8 | 3 | 11 | N/A | N/A | N/A | N/A |
| Otake et. Al. 2024 | 1 | 5 | 4 | 9 | N/A | N/A | N/A | N/A | N/A | N/A |
| Frey et. Al. 2000 | 69 | 77 | N/A | N/A | 13 | 15 | N/A | N/A | N/A | N/A |
| Gaster et. Al. 2003 | 24 | 29 | 8 | 3 | 2 | 4 | N/A | N/A | N/A | N/A |
| Gil et. Al. 2007 | N/A | N/A | N/A | N/A | N/A | N/A | N/A | N/A | N/A | N/A |
| Chieffo et. Al. 2013 | N/A | N/A | N/A | N/A | N/A | N/A | N/A | N/A | N/A | N/A |
| Yoon et. Al. 2013 | 7 | 18 | 22 | 27 | N/A | N/A | N/A | N/A | N/A | N/A |
| Ueki et. Al. 2020 | 4 | 2 | 6 | 3 | N/A | N/A | N/A | N/A | N/A | N/A |
| Zhang et. Al. 2018 | 67 | 86 | 126 | 144 | 10 | 8 | 99 | 115 | 245 | 244 |
| Hong et. Al. 2015 | 34 | 29 | 76 | 69 | 20 | 16 | N/A | N/A | N/A | N/A |
| Tan et. Al. 2015 | 10 | 13 | N/A | N/A | N/A | N/A | N/A | N/A | N/A | N/A |
| Stables et. Al. 2022 | 117 | 129 | 147 | 140 | N/A | N/A | N/A | N/A | N/A | N/A |
| Tian et. Al. 2015 | N/A | N/A | N/A | N/A | N/A | N/A | N/A | N/A | N/A | N/A |
| Götberg et. Al. 2017 | 337 | 335 | 429 | 425 | 49 | 43 | N/A | N/A | N/A | N/A |
| Maznyczka et. Al. 2023 | 57 | 69 | 75 | 116 | N/A | N/A | 51 | 64 | 11 | 10 |
| Russo et. Al. 2009 | 138 | 118 | 95 | 102 | 71 | 81 | N/A | N/A | N/A | N/A |
| Rioufol et. Al. 2021 | 90 | 100 | 115 | 127 | N/A | N/A | N/A | N/A | 188 | 180 |
| Lee et. Al. 2024 | 47 | 56 | 117 | 119 | 7 | 6 | N/A | N/A | N/A | 40 |
| Park et. Al. 2015 | 22 | 20 | 22 | 20 | N/A | N/A | N/A | N/A | N/A | N/A |
| Oemrawsingh et. Al. 2003 | N/A | N/A | N/A | N/A | N/A | N/A | N/A | N/A | N/A | N/A |
| Amabile et. Al. 2025 | N/A | N/A | 18 | 25 | 2 | 0 | N/A | N/A | 4 | 2 |
| Mariani et. Al. 2014 | N/A | N/A | 11 | 5 | 6 | 7 | N/A | N/A | 20 | 17 |
| Holm et. Al. 2023 | 170 | 180 | 244 | 257 | 7 | 9 | N/A | N/A | 12 | 14 |
| Ali et. Al. 2023 | 252 | 303 | 160 | 166 | 63 | 53 | N/A | N/A | 198 | 235 |
| Puymirat et. Al. 2021 | 45 | 31 | 59 | 44 | N/A | N/A | 37 | 29 | 11 | 12 |
| Davies et. Al. 2017 | 358 | 376 | 489 | 527 | N/A | N/A | 77 | 67 | N/A | N/A |
| Tonino et. Al. 2009 | 187 | 180 | 146 | 129 | N/A | N/A | N/A | N/A | N/A | N/A |
| Mudra et. Al. 2001 | 87 | 89 | 55 | 55 | 8 | 11 | N/A | N/A | N/A | N/A |
| Muramatsu et et. Al. 2020 | 19 | 16 | 24 | 26 | 0 | 0 | N/A | N/A | N/A | N/A |
| Vasiljevs et. Al. 2023 | N/A | N/A | N/A | N/A | N/A | N/A | 6 | 3 | N/A | N/A |
| Jia et. Al. 2025 | N/A | N/A | N/A | N/A | N/A | N/A | N/A | N/A | N/A | N/A |
| Kim et et. Al. 2015 | 16 | 16 | 31 | 32 | 3 | 5 | 12 | 10 | N/A | N/A |
| Kang et. Al. 2023 | 78 | 63 | 226 | 202 | 33 | 18 | 30 | 16 | 20 | 26 |
| Escaned et. Al. 2024 | N/A | N/A | N/A | N/A | N/A | N/A | N/A | N/A | N/A | N/A |
| Amabile et. Al. 2025 | N/A | N/A | 18 | 25 | 2 | 0 | N/A | N/A | 4 | 2 |
| 3-C. Clinical Presentation at Index PCI | | | | | | | | | | |
| Author / year | Unstable Angina | | Stable Angina | | STEMI | | NSTEMI | |  | |
|  | Modality | Control Modality | Modality | Control Modality | Modality | Control Modality | Modality | Control Modality |  |  |
| Li et. Al. 2024 | 699 | 726 | 570 | 537 | 484 | 489 | N/A | N/A |  |  |
| Yang et. Al. 2025 | N/A | N/A | 519 | 544 | 4 | 4 | 12 | 15 |  |  |
| Stone et. Al. 2025 | 238 | 180 | 1008 | 579 | N/A | N/A | N/A | N/A |  |  |
| Quintella et. Al. 2019 | 42 | 27 | N/A | N/A | N/A | N/A | N/A | N/A |  |  |
| Liu et. Al. 2019 | 3 | 4 | 20 | 18 | 127 | 126 | 17 | 21 |  |  |
| Burzotta et. Al. 2020 | N/A | N/A | N/A | N/A | N/A | N/A | N/A | N/A |  |  |
| Jakabcin et. Al. 2010 | 42 | 40 | 41 | 45 | 22 | 30 | N/A | N/A |  |  |
| Groenland et. Al. 2025 | 17 | 26 | 39 | 48 | 89 | 72 | N/A | N/A |  |  |
| Chamie et. Al. 2021 | 22 | 18 | 20 | 22 | 9 | 10 | N/A | N/A |  |  |
| Chen et. Al. 2015 | 99 | 98 | 36 | 34 | 6 | 14 | 19 | 14 |  |  |
| Zhang et. Al. 2016 | N/A | N/A | N/A | N/A | N/A | N/A | N/A | N/A |  |  |
| Wang et. Al. 2015 | N/A | N/A | N/A | N/A | N/A | N/A | N/A | N/A |  |  |
| Lee et. Al. 2023 | N/A | N/A | 131 | 134 | 153 | 144 | 157 | 188 |  |  |
| Ali et. Al. 2025 | 82 | 89 | 157 | 174 | 167 | 157 | 110 | 85 |  |  |
| Layland et. Al. 2015 | N/A | N/A | N/A | N/A | N/A | N/A | N/A | N/A |  |  |
| Kubo et. Al. 2017 | 363 | 352 | 48 | 53 | N/A | N/A | N/A | N/A |  |  |
| Ali et. Al. 2016 | 25 | 33 | 54 | 49 | 6 | 6 | 19 | 19 |  |  |
| Otake et. Al. 2024 | N/A | N/A | N/A | N/A | 38 | 35 | 19 | 20 |  |  |
| Frey et. Al. 2000 | 15 | 27 | N/A | N/A | N/A | N/A | N/A | N/A |  |  |
| Gaster et. Al. 2003 | N/A | N/A | N/A | N/A | N/A | N/A | N/A | N/A |  |  |
| Gil et. Al. 2007 | N/A | N/A | 80 | 83 | N/A | N/A | N/A | N/A |  |  |
| Chieffo et. Al. 2013 | 42 | 37 | N/A | N/A | N/A | N/A | N/A | N/A |  |  |
| Yoon et. Al. 2013 | N/A | N/A | N/A | N/A | N/A | N/A | N/A | N/A |  |  |
| Ueki et. Al. 2020 | 4 | 9 | 4 | 7 | 2 | 5 | N/A | N/A |  |  |
| Zhang et. Al. 2018 | 60 | 61 | 95 | 96 | 488 | 466 | 81 | 101 |  |  |
| Hong et. Al. 2015 | 242 | 356 | 358 | 356 | N/A | N/A | N/A | N/A |  |  |
| Tan et. Al. 2015 | 43 | 41 | 18 | 21 | N/A | N/A | N/A | N/A |  |  |
| Stables et. Al. 2022 | N/A | N/A | N/A | N/A | N/A | N/A | N/A | N/A |  |  |
| Tian et. Al. 2015 | N/A | N/A | N/A | N/A | N/A | N/A | N/A | N/A |  |  |
| Götberg et. Al. 2017 | 211 | 208 | 632 | 632 | N/A | N/A | 176 | 178 |  |  |
| Maznyczka et. Al. 2023 | 92 | 80.2 | 134.8 | 145.7 | N/A | N/A | N/A | N/A |  |  |
| Russo et. Al. 2009 | N/A | N/A | N/A | N/A | N/A | N/A | N/A | N/A |  |  |
| Rioufol et. Al. 2021 | N/A | N/A | 89 | 102 | N/A | N/A | N/A | N/A |  |  |
| Lee et. Al. 2024 | N/A | N/A | N/A | N/A | 53 | 56 | 166 | 171 |  |  |
| Park et. Al. 2015 | N/A | N/A | 52 | 54 | N/A | N/A | N/A | N/A |  |  |
| Oemrawsingh et. Al. 2003 | N/A | N/A | N/A | N/A | N/A | N/A | N/A | N/A |  |  |
| Amabile et. Al. 2025 | N/A | N/A | N/A | N/A | N/A | N/A | N/A | N/A |  |  |
| Mariani et. Al. 2014 | N/A | N/A | 31 | 30 | N/A | N/A | N/A | N/A |  |  |
| Holm et. Al. 2023 | 53 | 58 | 330 | 321 | N/A | N/A | 79 | 78 |  |  |
| Ali et. Al. 2023 | 355 | 331 | 333 | 358 | 68 | 73 | 304 | 299 |  |  |
| Puymirat et. Al. 2021 | N/A | N/A | N/A | N/A | N/A | N/A | N/A | N/A |  |  |
| Davies et. Al. 2017 | N/A | N/A | N/A | N/A | 49 | 42 | 137 | 142 |  |  |
| Tonino et. Al. 2009 | N/A | N/A | N/A | N/A | N/A | N/A | N/A | N/A |  |  |
| Mudra et. Al. 2001 | 98 | 89 | N/A | N/A | N/A | N/A | N/A | N/A |  |  |
| Muramatsu et et. Al. 2020 | N/A | N/A | N/A | N/A | N/A | N/A | N/A | N/A |  |  |
| Vasiljevs et. Al. 2023 | N/A | N/A | N/A | N/A | 4 | 1 | 5 | 7 |  |  |
| Jia et. Al. 2025 | 46 | 47 | N/A | N/A | 6 | 8 | 4 | 4 |  |  |
| Kim et et. Al. 2015 | N/A | N/A | N/A | N/A | N/A | N/A | N/A | N/A |  |  |
| Kang et. Al. 2023 | 137 | 135 | 663 | 654 | 0 | 0 | 99 | 99 |  |  |
| Escaned et. Al. 2024 | N/A | N/A | N/A | N/A | N/A | N/A | N/A | N/A |  |  |
| Amabile et. Al. 2025 | N/A | N/A | N/A | N/A | N/A | N/A | N/A | N/A |  |  |
| 3-D. Coronary Anatomy | | | | | | | | | | |
| Author / year | SVD | | DVD | | TVD | |  |  |  |  |
|  | Modality | Control Modality | Modality | Control Modality | Modality | Control Modality |  |  |  |  |
| Li et. Al. 2024 | N/A | N/A | N/A | N/A | N/A | N/A |  |  |  |  |
| Yang et. Al. 2025 | 378 | 398 | 295 | 273 | 150 | 157 |  |  |  |  |
| Stone et. Al. 2025 | N/A | N/A | N/A | N/A | N/A | N/A |  |  |  |  |
| Quintella et. Al. 2019 | N/A | N/A | N/A | N/A | N/A | N/A |  |  |  |  |
| Liu et. Al. 2019 | N/A | N/A | N/A | N/A | N/A | N/A |  |  |  |  |
| Burzotta et. Al. 2020 | N/A | N/A | N/A | N/A | N/A | N/A |  |  |  |  |
| Jakabcin et. Al. 2010 | 42 | 48 | 33 | 30 | 30 | 26 |  |  |  |  |
| Groenland et. Al. 2025 | N/A | N/A | N/A | N/A | N/A | N/A |  |  |  |  |
| Chamie et. Al. 2021 | N/A | N/A | N/A | N/A | N/A | N/A |  |  |  |  |
| Chen et. Al. 2015 | 50 | 48 | 52 | 48 | 58 | 64 |  |  |  |  |
| Zhang et. Al. 2016 | N/A | N/A | N/A | N/A | N/A | N/A |  |  |  |  |
| Wang et. Al. 2015 | N/A | N/A | N/A | N/A | N/A | N/A |  |  |  |  |
| Lee et. Al. 2023 | 127 | 90 | N/A | N/A | N/A | N/A |  |  |  |  |
| Ali et. Al. 2025 | 28 | 33 | N/A | N/A | N/A | N/A |  |  |  |  |
| Layland et. Al. 2015 | 62 | 68 | 72 | 69 | 33 | 27 |  |  |  |  |
| Kubo et. Al. 2017 | N/A | N/A | N/A | N/A | N/A | N/A |  |  |  |  |
| Ali et. Al. 2016 | N/A | N/A | N/A | N/A | N/A | N/A |  |  |  |  |
| Otake et. Al. 2024 | 11 | 11 | N/A | N/A | N/A | N/A |  |  |  |  |
| Frey et. Al. 2000 | N/A | N/A | N/A | N/A | N/A | N/A |  |  |  |  |
| Gaster et. Al. 2003 | 26 | 25 | 23 | 22 | 5 | 7 |  |  |  |  |
| Gil et. Al. 2007 | N/A | N/A | N/A | N/A | N/A | N/A |  |  |  |  |
| Chieffo et. Al. 2013 | N/A | N/A | N/A | N/A | N/A | N/A |  |  |  |  |
| Yoon et. Al. 2013 | N/A | N/A | N/A | N/A | N/A | N/A |  |  |  |  |
| Ueki et. Al. 2020 | 18 | 19 | 1 | 2 | N/A | N/A |  |  |  |  |
| Zhang et. Al. 2018 | 381 | 414 | N/A | N/A | N/A | N/A |  |  |  |  |
| Hong et. Al. 2015 | 230 | 210 | 256 | 260 | 214 | 230 |  |  |  |  |
| Tan et. Al. 2015 | 14 | 13 | 24 | 22 | 16 | 17 |  |  |  |  |
| Stables et. Al. 2022 | 218 | 265 | 112 | 108 | 62 | 36 |  |  |  |  |
| Tian et. Al. 2015 | N/A | N/A | N/A | N/A | N/A | N/A |  |  |  |  |
| Götberg et. Al. 2017 | 452 | 453 | 256 | 267 | 108 | 101 |  |  |  |  |
| Maznyczka et. Al. 2023 | N/A | N/A | N/A | N/A | N/A | N/A |  |  |  |  |
| Russo et. Al. 2009 | N/A | N/A | N/A | N/A | N/A | N/A |  |  |  |  |
| Rioufol et. Al. 2021 | 12 | 13 | 201 | 223 | 247 | 231 |  |  |  |  |
| Lee et. Al. 2024 | N/A | N/A | N/A | N/A | 383 | 381 |  |  |  |  |
| Park et. Al. 2015 | 42 | 49 | 49 | 39 | 23 | 27 |  |  |  |  |
| Oemrawsingh et. Al. 2003 | N/A | N/A | N/A | N/A | N/A | N/A |  |  |  |  |
| Amabile et. Al. 2025 | N/A | N/A | N/A | N/A | N/A | N/A |  |  |  |  |
| Mariani et. Al. 2014 | N/A | N/A | N/A | N/A | N/A | N/A |  |  |  |  |
| Holm et. Al. 2023 | N/A | N/A | N/A | N/A | N/A | N/A |  |  |  |  |
| Ali et. Al. 2023 | N/A | N/A | N/A | N/A | N/A | N/A |  |  |  |  |
| Puymirat et. Al. 2021 | 7 | 11 | 424 | 447 | 151 | 115 |  |  |  |  |
| Davies et. Al. 2017 | N/A | N/A | N/A | N/A | N/A | N/A |  |  |  |  |
| Tonino et. Al. 2009 | N/A | N/A | N/A | N/A | N/A | N/A |  |  |  |  |
| Mudra et. Al. 2001 | N/A | N/A | N/A | N/A | N/A | N/A |  |  |  |  |
| Muramatsu et et. Al. 2020 | 34 | 31 | 13 | 18 | 7 | 6 |  |  |  |  |
| Vasiljevs et. Al. 2023 | N/A | N/A | N/A | N/A | N/A | N/A |  |  |  |  |
| Jia et. Al. 2025 | N/A | N/A | N/A | N/A | N/A | N/A |  |  |  |  |
| Kim et et. Al. 2015 | 57 | 75 | 66 | 64 | 78 | 62 |  |  |  |  |
| Kang et. Al. 2023 | 397 | 374 | 350 | 346 | 258 | 283 |  |  |  |  |
| Escaned et. Al. 2024 | N/A | N/A | N/A | N/A | N/A | N/A |  |  |  |  |
| Amabile et. Al. 2025 | N/A | N/A | N/A | N/A | N/A | N/A |  |  |  |  |
| 3-E. Lesion & Procedural Characteristics | | | | | | | | | | |
| Author / year | Lesions | | Bifurcation | | Left Main Stenosis | | Complete Total Occlusion | | Small Vessel Disease | |
|  | Modality | Control Modality | Modality | Control Modality | Modality | Control Modality | Modality | Control Modality | Modality | Control Modality |
| Li et. Al. 2024 | N/A | N/A | N/A | N/A | N/A | N/A | N/A | N/A | N/A | N/A |
| Yang et. Al. 2025 | 919 | 901 | N/A | N/A | N/A | N/A | N/A | N/A | N/A | N/A |
| Stone et. Al. 2025 | 1549 | 943 | 10 | 11 | 10 | 11 | N/A | N/A | N/A | N/A |
| Quintella et. Al. 2019 | N/A | N/A | N/A | N/A | N/A | N/A | N/A | N/A | N/A | N/A |
| Liu et. Al. 2019 | 167 | 169 | 20 | 22 | 167 | 169 | 20 | 22 | N/A | N/A |
| Burzotta et. Al. 2020 | N/A | N/A | N/A | N/A | N/A | N/A | N/A | N/A | N/A | N/A |
| Jakabcin et. Al. 2010 | 125 | 129 | N/A | N/A | 4 | 3 | N/A | N/A | N/A | N/A |
| Groenland et. Al. 2025 | N/A | N/A | N/A | N/A | N/A | N/A | 5 | 19 | N/A | N/A |
| Chamie et. Al. 2021 | 51 | 52 | N/A | N/A | N/A | N/A | N/A | N/A | N/A | N/A |
| Chen et. Al. 2015 | 14 | 15 | N/A | N/A | N/A | N/A | 13 | 14 | N/A | N/A |
| Zhang et. Al. 2016 | N/A | N/A | N/A | N/A | N/A | N/A | N/A | N/A | N/A | N/A |
| Wang et. Al. 2015 | 4 | 2 | 4 | 2 | N/A | N/A | N/A | N/A | N/A | N/A |
| Lee et. Al. 2023 | N/A | N/A | N/A | N/A | N/A | N/A | N/A | N/A | N/A | N/A |
| Ali et. Al. 2025 | 553 | 546 | 18 | 15 | N/A | N/A | N/A | N/A | N/A | N/A |
| Layland et. Al. 2015 | 5 | 4 | N/A | N/A | N/A | N/A | N/A | N/A | N/A | N/A |
| Kubo et. Al. 2017 | 409 | 400 | 154 | 157 | N/A | N/A | N/A | N/A | N/A | N/A |
| Ali et. Al. 2016 | N/A | N/A | N/A | N/A | N/A | N/A | N/A | N/A | N/A | N/A |
| Otake et. Al. 2024 | N/A | N/A | N/A | N/A | N/A | N/A | 28 | 25 | N/A | N/A |
| Frey et. Al. 2000 | 166 | 190 | N/A | N/A | N/A | N/A | N/A | N/A | N/A | N/A |
| Gaster et. Al. 2003 | 54 | 54 | N/A | N/A | N/A | N/A | N/A | N/A | N/A | N/A |
| Gil et. Al. 2007 | N/A | N/A | N/A | N/A | N/A | N/A | N/A | N/A | N/A | N/A |
| Chieffo et. Al. 2013 | 182 | 179 | N/A | N/A | N/A | N/A | 25 | 32 | 40 | 42 |
| Yoon et. Al. 2013 | 735 | 1165 | N/A | N/A | N/A | N/A | N/A | N/A | N/A | N/A |
| Ueki et. Al. 2020 | N/A | 21 | 20 | N/A | N/A | N/A | N/A | N/A | N/A | N/A |
| Zhang et. Al. 2018 | 962 | 1016 | N/A | N/A | N/A | N/A | N/A | N/A | N/A | N/A |
| Hong et. Al. 2015 | N/A | N/A | N/A | N/A | N/A | N/A | N/A | N/A | N/A | N/A |
| Tan et. Al. 2015 | N/A | N/A | 32 | 34 | 61 | 62 | N/A | N/A | N/A | N/A |
| Stables et. Al. 2022 | N/A | N/A | N/A | N/A | 43 | 48 | N/A | N/A | N/A | N/A |
| Tian et. Al. 2015 | N/A | N/A | N/A | N/A | N/A | N/A | N/A | N/A | N/A | N/A |
| Götberg et. Al. 2017 | 1568 | 1436 | N/A | N/A | N/A | N/A | N/A | N/A | N/A | N/A |
| Maznyczka et. Al. 2023 | N/A | N/A | 179 | 276 | N/A | N/A | N/A | N/A | N/A | N/A |
| Russo et. Al. 2009 | N/A | N/A | 3 | 2 | 3 | 2 | N/A | N/A | N/A | N/A |
| Rioufol et. Al. 2021 | 1632 | 1634 | 58 | 50 | N/A | N/A | 63 | 55 | N/A | N/A |
| Lee et. Al. 2024 | N/A | N/A | 118 | 114 | N/A | N/A | N/A | N/A | N/A | N/A |
| Park et. Al. 2015 | 114 | 115 | N/A | N/A | N/A | N/A | N/A | N/A | N/A | N/A |
| Oemrawsingh et. Al. 2003 | N/A | N/A | N/A | N/A | N/A | N/A | N/A | N/A | N/A | N/A |
| Amabile et. Al. 2025 | N/A | N/A | 4 | 5 | N/A | N/A | N/A | N/A | N/A | N/A |
| Mariani et. Al. 2014 | N/A | N/A | 10 | 11 | 2 | 3 | N/A | N/A | 12 | 17 |
| Holm et. Al. 2023 | N/A | N/A | 111 | 116 | 111 | 116 | N/A | N/A | N/A | N/A |
| Ali et. Al. 2023 | 1353 | 1409 | N/A | N/A | N/A | N/A | 94 | 79 | N/A | N/A |
| Puymirat et. Al. 2021 | 718 | 706 | N/A | N/A | 3 | 4 | N/A | N/A | N/A | N/A |
| Davies et. Al. 2017 | 451 | 557 | N/A | N/A | N/A | N/A | N/A | N/A | N/A | N/A |
| Tonino et. Al. 2009 | 1414 | 1350 | N/A | N/A | N/A | N/A | 54 | 37 | N/A | N/A |
| Mudra et. Al. 2001 | N/A | N/A | N/A | N/A | N/A | N/A | N/A | N/A | N/A | N/A |
| Muramatsu et et. Al. 2020 | 62 | 64 | N/A | N/A | N/A | N/A | N/A | N/A | N/A | N/A |
| Vasiljevs et. Al. 2023 | N/A | N/A | N/A | N/A | N/A | N/A | N/A | N/A | N/A | N/A |
| Jia et. Al. 2025 | N/A | N/A | N/A | N/A | N/A | N/A | N/A | N/A | N/A | N/A |
| Kim et et. Al. 2015 | 201 | 201 | N/A | N/A | N/A | N/A | N/A | N/A | N/A | N/A |
| Kang et. Al. 2023 | 1307 | 1404 | 116 | 148 | 116 | 148 | 56 | 52 | N/A | N/A |
| Escaned et. Al. 2024 | N/A | N/A | N/A | N/A | N/A | N/A | N/A | N/A | N/A | N/A |
| Amabile et. Al. 2025 | N/A | N/A | 4 | 5 | N/A | N/A | N/A | N/A | N/A | N/A |
